# Lipidomics-based association study reveals genomic signatures of anti-cancer qualities of pigmented rice sprouts

**DOI:** 10.3389/fpls.2025.1533442

**Published:** 2025-01-28

**Authors:** Rhowell Navarro Tiozon, Erstelle Pasion-Uy, Saleh Alseekh, Kristel June D. Sartagoda, Shem Gempesaw, Joel H. G. Tolentino, Alisdair R. Fernie, Nese Sreenivasulu

**Affiliations:** ^1^ Consumer-driven Grain Quality and Nutrition Center, Strategic Innovation Platform, International Rice Research Institute, Los Baños, Philippines; ^2^ Max-Planck-Institute of Molecular Plant Physiology, Potsdam-Golm, Germany; ^3^ Center of Plant Systems Biology and Biotechnology, Plovdiv, Bulgaria; ^4^ Department of Food Science and Chemistry, College of Science and Mathematics, University of the Philippines Mindanao, Davao City, Philippines

**Keywords:** antioxidants, anticancer, lipidomics, lipase, pigmented rice

## Abstract

**Introduction:**

The genetic wealth present in pigmented rice varieties offer abundant variation in different sources of antioxidants to meet nutritional security targets among rice-consuming communities. There is limited knowledge of the dynamic changes in the lipidome of rice during germination and the corresponding genes associated with the antioxidant and anti-cancerous properties of lipophilic fractions of pigmented rice sprouts (PRS).

**Methods:**

In this study, we profiled the lipidome of diverse pigmented rice collections of germinated sprouts. Further, we employed Genome-wide association studies (GWAS), gene-set analysis, and targeted association analysis to identify the candidate genes linked to these lipids.

**Results:**

The genetic analyses revealed 72 candidate genes involved in the regulation of these accumulating lipids in PRS. Marker trait associations (MTA) analysis shown that the combination GGTAAC/ACAAGCTGGGCCC was associated with increased levels of unsaturated lipids and carotenoids, which likely underlie these beneficial effects. This superior MTA combination exhibited potent inhibitory activity against HCT116 and A549 cell lines, with average 1/IC50 values of 0.03 and 0.02 (mL/μg), respectively, compared to the inferior MTAs.

**Discussion:**

Collectively, our findings demonstrate that MTAs linked to selected GDSL esterase/lipase (GELP) genes, OsACP1, and lecithin-cholesterol acyltransferase significantly enhance antioxidant and anti-cancer properties, potentially through the mobilization of unsaturated lipids and carotenoids during germination. This study offers valuable insights into the health-promoting potential of germinated rice sprouts as a rich dietary source of antioxidants beneficial to human health.

## 1 Introduction

Rice holds a prominent position among the world’s most essential crops as it serves as the primary staple food for more than half of the global population, primarily due to its high starch content. Apart from starch, lipids are also present in whole grain rice, constituting around 3-4% of the overall grain composition (Cai et al., 2020). Rice lipids that are concentrated in the bran mostly include triacylglycerols (TAGs), which constitute free fatty acids such as palmitic (16:0), oleic (18:1), and linoleic (18:2) acids. The accumulation of TAGs in rice grains occurs rapidly within the period of 5 to 12 days after fertilization (Ichihara et al., 2003). There is a significant variation observed for 11 oil-related traits, and the oil composition of rice grains differed among the subpopulations (Zhou et al., 2021). Furthermore, there was significant variation in fatty acid content within a core set of 190 Indian rice landraces (Sahu et al., 2020). Recently, a number of investigations have employed genome-wide association studies (GWAS) to identify genetic factors associated with the biosynthesis of fatty acids in grains (Zhiguo et al., 2019; Zhou et al., 2021; Mai et al., 2023). Zhou et al (2021) identified genes, such as *PAL6, LIN6, MYR2*, and *ARA6* that play a role in the natural variation of fatty acids across subpopulations. Concurrently, GWAS has revealed significant QTL and genes that regulate the fatty acid composition in rice bran oil from Vietnamese rice landraces (Mai et al., 2023). From a nutritional standpoint, rice lipids are known to confer various health benefits to humans, such as scavenging free radicals, bolstering the immune system, and mitigating the likelihood of developing cancer and cardiovascular disease (Tong and Bao, 2019). Taken together, it is therefore imperative to conduct an analysis of the lipidome of rice and ascertain its potential dietary significance.

Seed germination is a vital and intricate stage in the life cycle characterized by dynamic yet synchronized processes that enable the transformation of quiescent embryonic cells into an actively metabolizing state (Cai et al., 2020; Tiozon et al., 2023). The mobilization of storage lipids during germination commences with the hydrolysis of triacylglycerols in oleosomes by lipases into free fatty acids and glycerol (Sinha et al., 2020). The fatty acids undergo β-oxidation within peroxisomes, followed by partial progression of the glyoxylate cycle in both the peroxisome and cytoplasmic compartments. The culminating phase of germination encompasses gluconeogenesis and the production of smaller monosaccharides that serve as a means of energy reserves (Bansal et al., 2021). Given that germination induces modifications in the lipid profile through the mobilization of triacylglycerols (TAGs), it is pertinent to investigate the alterations in the rice lipidome resulting from this process, to identify lipid molecules exhibiting novel health benefits for human consumption. Through multi-omics strategy, genes responsible in the production of flavonoid glycosides in germinating seeds were identified (Tiozon et al., 2023). Previously, we provided a detailed characterization of the lipidome of pigmented rice sprouts (PRS), highlighting their abundance in health-promoting lipids (Tiozon et al., 2024). However, studies utilizing genome-wide association studies (GWAS) to explore the lipidome of PRS and its correlation with antioxidant and anticancer properties remain scarce, leaving a critical gap in understanding the genetic regulation and health implications of these bioactive compounds.

While the direct consumption of germinating seeds is limited, analyzing the lipid profile and its health beneficial factors contributing to antioxidant and anti-cancerous activities during germination provides valuable insights into the metabolic pathways and regulatory mechanisms that influence the final lipid composition of the sprouts, which could be positioned as functional food in future. These pathways, such as fatty acid biosynthesis, desaturation, and modification, are crucial in determining the types and levels of bioactive lipids, including unsaturated fatty acids and carotenoids that ultimately contribute to the anti-cancer properties of the PRS and its novel sources of genetic variability in the whole grains. Hence, the present work aimed to (i) investigate the dietary properties of these lipids, including their antioxidant and anticancer activities in PRS and compared with whole grain; (ii) identify candidate genes involved in lipid accumulation and degradation during seed germination through a combination of lipidomic analysis and genome-wide association studies (GWAS), while also exploring potential selective sweeps of target genes associated with domestication; and (iii) determine superior rice lines based on MTAs to facilitate the breeding of rice varieties with enhanced levels of health-beneficial lipids in PRS.

## 2 Materials and methods

### 2.1 Sample preparation

A diversity set comprising 293 samples of pigmented rice was examined, including 18 purple-colored, 256 variable-purple-colored, 16 red-colored, and 3 light brown varieties (**Supplementary Table S1**). These samples were selected from the International Rice Research Institute (IRRI) genebank, purified them through single seed dissent and were planted during the 2019 dry season at the experimental station of IRRI in Los Baños, Laguna, the Philippines by following the standard agronomic practices and irrigated conditions. From the harvested paddy samples of pigmented rice core collection, the state of dormancy was terminated by subjecting rice seeds to a temperature of 50°C for duration of five days. The germination process was initiated in accordance with the described method (Cáceres et al., 2017; Tiozon et al., 2023).

### 2.2 Extraction procedure and data processing for lipidomic analysis

The extraction protocol and data process followed the previous method (Tiozon et al., 2024). Briefly, 50 mg of PRS samples were extracted with 1.2 mL methyl tert-butyl ether:methanol, 500 µL of the upper lipid-containing phase was dried in a speedVac concentrator and re-suspended in 250 µL acetonitrile: 2-propanol (7:3, v/v) solution. The workflow included peak detection, retention time alignment, and removal of chemical noise. Identified lipids were confirmed by manual verification of the chromatograms using Xcalibur (version 3.0, Thermo-Fisher, Bremen, Germany). The mass spectra were acquired using an Orbitrap high-resolution mass spectrometer: Fourier-transform mass spectrometer (FT-MS) coupled with a linear ion trap (LTQ) Orbitrap XL (ThermoFisher Scientific, https://www.thermofisher.com) (**Supplementary Tables S2**, **S3**) (Alseekh et al., 2021). Multiple multivariate statistical methods were employed to investigate the variability of the lipidome pattern in germinated rice seeds. To this end, principal component analysis (PCA), partial least squares-discriminant analysis (PLS-DA), variable importance in projection scores, heatmap, and pathway enrichment analysis for data visualization were performed using MetaboAnalyst 4.0 software (Chong et al., 2019). The fold change (log2FC) comparison of lipids was assessed between different color classifications among germinated sprouts (**Supplementary Table S4**). The lipophilicity of the lipid groups was estimated based on the LIPID MAPS comprehensive classification system for lipids (Fahy et al., 2009).

### 2.3 Measurement of antioxidant capacity

The PRS samples subjected to DPPH (2,2-Diphenyl-1-picrylhydrazyl) radical scavenging activity and ABTS (2,2’-azino-bis(3-ethylbenzothiazoline-6-sulfonic acid)) assays according to Chu et al. (2020), and the Ferric Reducing Antioxidant Power (FRAP) assay was performed following the previous methods (Tomasina et al., 2012; Chu et al., 2020). The absorbance was measured using a microplate reader (BMG SPECTROstar Nano) at 515 nm for DPPH, 734 nm for ABTS, and 620 nm for the FRAP assay. The results were expressed as mg Trolox equivalent per g of sample. All calibration curves used have R^2^ = 0.999. Correlation analysis was performed to assess the contribution of various lipid compounds to the antioxidant properties of PRS.

### 2.4 MTT (3-(4,5-Dimethylthiazol-2-yl)-2,5-Diphenyltetrazolium Bromide) assay for cell viability and proliferation

The bioactivity of samples was tested against colon carcinoma (HCT116) and lung adenocarcinoma (A549) cell lines (American Type Culture Collection, Manassas, VA, USA) following the previous method with modifications (Brotman et al., 2021). The cell line was maintained and cultured using Dulbecco’s Modified Eagle Medium (DMEM) supplemented with 10% heat-inactivated fetal bovine serum and 1% penicillin-streptomycin solution (Gibco, Life Technologies Corporation, New York, United States). The cells were grown in an incubator set at a temperature of 37°C in a 5% CO_2_ humidified atmosphere (rh=95%).

Flasks containing cells of >70% confluency were trypsinized and the cells were harvested to obtain a cell suspension containing 50,500 cells/mL. About 198 µL of the cell suspension was transferred into a sterile 96-well microplate and incubated overnight at similar culture conditions. After overnight incubation, 2 µL of serially diluted samples (lipophilic extracts of PRS from superior and inferior MTAs) in DMSO were added into six designated wells, resulting in the extracts’ final concentrations of 240, 120, 60, 30, 15, and 7.5 µg/mL. Two-fold serial dilutions of doxorubicin (2 µg/mL) served as positive control and wells treated with DMSO served as solvent vehicle control. All treatments were performed in triplicate and independently repeated thrice. After 72 h of incubation, the spent medium in the wells was removed and 5 mg/mL MTT (Life Technologies Corporation, Eugene, Oregon) in phosphate buffer solution was added to each well. Plates were re-incubated for four hours and DMSO was added thereafter. The absorbance of each well was read at 570 nm using a microplate reader. The percent growth inhibition was calculated using the equation below. The half maximal inhibitory concentration (IC_50_) was computed based on the trendline of the sample concentrations between which 50% inhibitory activity falls.


$$\%\text{Inhibition}=(100-\frac{\text{Absorbance of sample-Absorbance of blank control}}{\text{Absorbance of vehicle control-Absorbance of blank control}})\text{x }100$$

### 2.5 Genetic analysis

The DNA was extracted from the developing seed of pigmented rice collection and the sequencing libraries were prepared using the previous method (Elshire et al., 2011). The SNP marker data were generated by genotyping by sequencing (GBS) and the variant calling of the pigmented rice lines was performed against the Nipponbare reference genome (MSUv7). The GBS data were screened based on ≥90% call rate, locus homozygosity, and minor allele frequency (MAF) ≥0.05 resulting to 459,826 high-quality biallelic SNPs. Genome-wide association studies (GWAS) based on the mixed linear model for single-locus analysis namely Efficient Mixed-Model Association eXpedited (EMMAX) were conducted using the rMVP (A Memory-efficient, Visualization-enhanced, and Parallel-accelerated tool) R package (Yin et al., 2021). Association of SNP to the lipid expression was considered significant at a Bonferroni correction threshold of *P* < 1.08736783x10^-07^ (or -log_10_(P) ≥ 6.96362352) using the formula 0.05/*m*, where *m* is the number of SNP markers utilized. Furthermore, the population structures were estimated based on the calculation of principal components and kinship matrices (**Supplementary Figure S1**). MAGMA software was utilized for gene-level analysis to identify significant genes identified based on Bonferroni-significant SNPs to further refine the list of candidates. The software includes internal correction for multiple testing, enabling the use of a significance threshold of *P* < 0.05 (De Leeuw et al., 2015). The heritability values of the traits were calculated using software tool called genome-wide complex trait analysis (GCTA) (Yang et al., 2011).

Haplotype blocks were examined using the blocks function implemented in PLINK 1.9, and the Haploview program was used to identify tag SNPs based on the threshold of the linkage disequilibrium (LD) coefficient (*D’*) > 0.8. SNPs associated with a P-value of <0.05 were considered significant and were used to generate the haplotypes (Barrett et al., 2005). Pairwise comparisons between alleles were based on the Mann-Whitney test and further confirmed by a t-test using the *ggstatplot* package in R (Patil, 2021). The genomic region, including 2 kb upstream of the start codon and 1 kb downstream of 3’ UTR was extracted using samtools (Danecek et al., 2021). Considering this region for each candidate gene identified using single-locus GWAS and known genes based on literature, targeted association analysis was performed through PLINK (Purcell et al., 2007) and EMMAX (Kang et al., 2010) for all traits of interest. Candidate genes with significant SNPs filtered at a 95% confidence level were visualized using Cytoscape (Shannon et al., 2003) and KnetMiner (Hassani-Pak et al., 2021). For the creation of marker-trait association (MTA), significant SNPs within each candidate gene were subjected to pruning (r^2^ < 0.2, window of 75 kb), and their contribution to phenotypic variance explained (PVE) was assessed using the *reml* function in LDAK. In cases where candidate genes were located within broad association peaks, SNP thinning was performed through LDAK’s heritability model, applying a threshold of r^2^ < 0.98 within a 75 kb window.

### 2.6 Variation in the population genomics parameters, gene duplication analysis, multiple sequence alignment, and phylogenetic analysis

The filtered GBS data with 459,826 high-quality biallelic SNPs was utilized to calculate the Tajima’s D index and the F_ST_ using TASSEL 5.2.87 and VCF tools using a 20-kb window with a step size of 5-kb for each subpopulation following the procedure of Danecek et al. (2011). The Tajima’s D value was calculated in a 20-kb non-overlapping window. The population structure was taken from previous analysis (Mbanjo et al., 2023). Both Tajima’s D index and the F_ST_ were analyzed genome-wide with the comparison between *Indica* and *Japonica.* Genetic regions containing the genes of interests were visualized in the context of selection sweeps. PLAZA 5.0 was employed to investigate gene duplications among the selected genes across different species (Van Bel et al., 2022). Utilizing protein sequences available in the Ensemble database (plants.ensembl.org/index.html), orthologs/homologs were identified through a BLAST search. Subsequently, the construction of a phylogenetic tree was performed using the MEGA X program, with the incorporation of 1,000 bootstrap replicates to gauge confidence levels at each node. Evolutionary distances were then computed using the Poisson correction method (Kumar et al., 2018).

## 3 Results

### 3.1 The lipophilic antioxidant properties of PRS lipids

**Figure 1:**
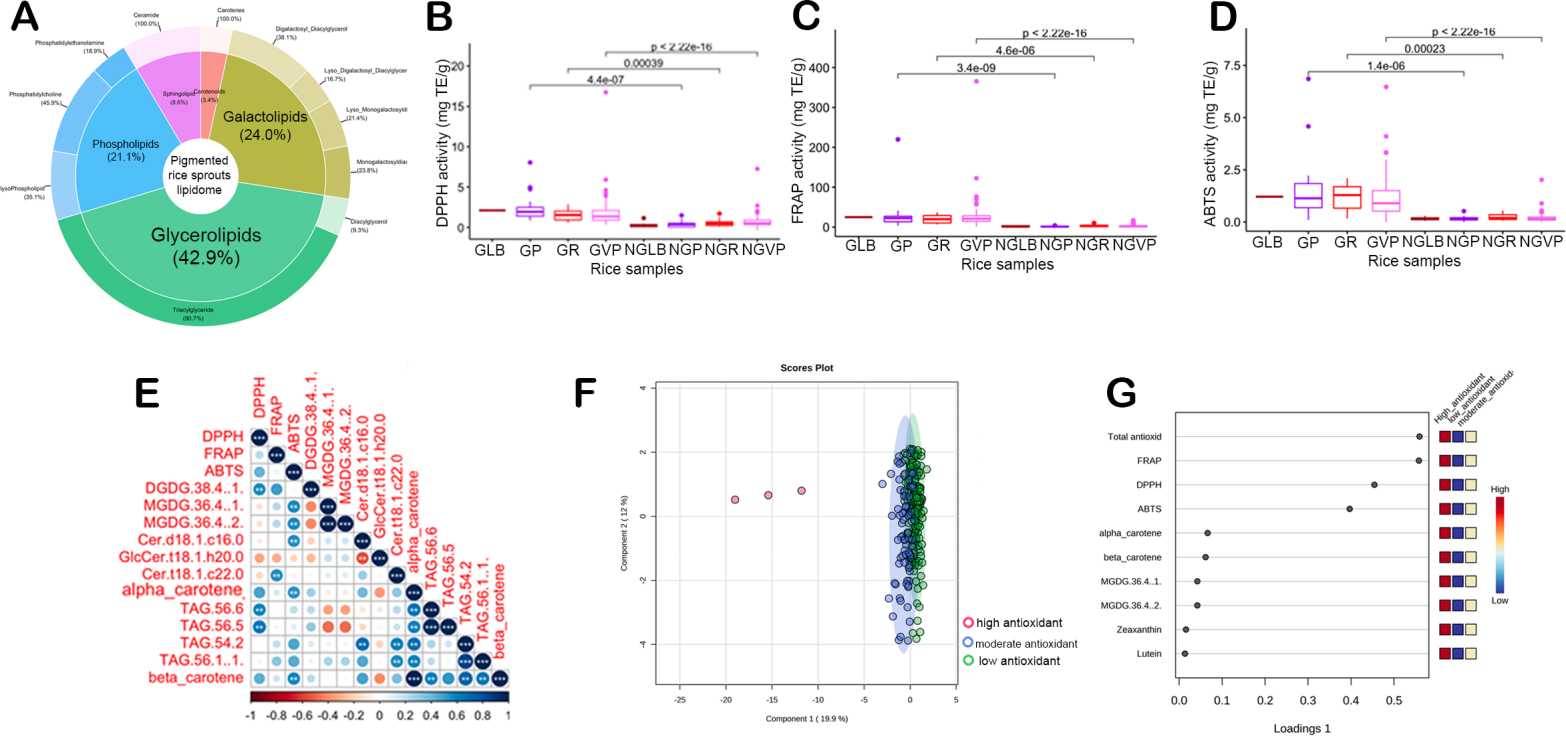
The lipidome and antioxidant properties of pigmented rice sprouts. **(A)** lipid composition of pigmented rice sprouts. **(B)** 2,2-diphenyl-1-picrylhydrazyl (DPPH) antioxidant, **(C)** ferric ion reducing antioxidant power (FRAP) antioxidant, **(D)** 2,2’-azino-bis(3-ethylbenzothiazoline-6-sulfonic acid (ABTS) antioxidant, **(E)** Correlation plot related to antioxidants. **(F)** Sparse Partial Least Squares Discriminant Analysis (sPLS-DA) discriminating the samples based on their antioxidant capacity. **(G)** Loading plots of important antioxidant parameters and lipids. In the boxplot, the solid middle line depicts the median, while the lower and upper whiskers signify the 25th and 75th percentiles, respectively. (GLB, Germinated Light Brown; GP, Germinated Purple; GR, Germinated Red; GVP, Germinated Variable Purple; NGLB, Non-germinated light brown; NGP, Non-germinated Purple; NGR, Non-germinated Red; NGVP, Non-Germinated Variable Purple; DPPH, 2,2-diphenyl-1-picrylhydrazyl; FRAP, Ferric Reducing Antioxidant Power; ABTS, 2,2’-azino-bis(3-ethylbenzothiazoline-6-sulfonic acid).

Lipidomic analysis of germinated rice sprouts revealed the major lipid classes – glycerolipids represented by diacylglycerols (DAG) and triacylglycerols (TAG) (42.9%), galactolipids comprised of lyso- counterparts, monogalactosyl diacylglycerols (MGDG), and digalactosyl diacylglycerols (DGDG) (24%), phospholipids consisting of lysophospolipids, phosphatidylcholines (PC), phosphatidylethanolamines (PE) (21.1%), sphingolipids (SL) (8.6%), and carotenoids (3.4%) (**Figure 1A**). **Supplementary Figure S2** shows the general structure of these lipid compounds. Changes in the accumulation of lipids with a higher degree of unsaturation appeared to be highly significant between PRS and non-pigmented sprouts (**Supplementary Figure S3A**). Moreover, significantly higher amounts of glycerolipids were observed in pigmented (i.e., purple, red, and variable purple) rice sprouts compared to non-pigmented rice (light-brown) based on average peak intensities of DAGs (**Supplementary Figure S3B**) and TAGs (**Supplementary Figure S3C**). Among glycerolipids, unsaturated diacylglycerols (DAGs; 36:2, 36:3, 34:2) and triacylglycerols (TAGs; 46:3, 50:4, 50:5, 54:4, 56:2, 56:3, 56:6, 58:3, and 58:4) were significantly more abundant in purple rice sprouts compared to non-pigmented samples. Notably, minimal lipid class differences were observed between light brown and variable purple rice, with the exception of slightly elevated levels of lutein, zeaxanthin, TAG 54:3, and TAG 56:3. Within PRS (**Supplementary Figures S3G-I**), carotenoid concentrations were higher in purple rice than in red rice. Furthermore, a consistent downregulation of certain sphingolipids and phosphatidylcholines was observed in PRS.

In general, the germinated sprouts lipophilic extracts exhibit higher antioxidant activity in comparison to mature grains (**Figures 1B-D**, **Supplementary Table S5**). The superior lines classified in germinated variable purple rice and germinated purple demonstrated remarkably higher antioxidant activities across all three antioxidant assays for lipophilic extractions. Results also showed a moderately strong positive correlation between the ABTS antioxidant and carotenoids such as alpha- and beta-carotene (**Figure 1E**). Among lipid fractions, MGDG 36:4 showed significant positive correlations with scavenging activity against the ABTS radical (**Figure 1E**). Specific triglycerides with higher degrees of unsaturation, such as TAG 56:6 and 56:5, and DGDG 38:4 showed significant positive correlations with the DPPH radical scavenging activity. On the other hand, DGDG 38:4 and Ceramide (Cer) (t18:0/22:0) were positively correlated with ferric reducing activity. Cluster analysis based on antioxidant values revealed distinct groups among the samples. Specifically, the sPLSDA demonstrated that three lines were black-colored rice samples—GBCR88, GBCR94, and GBCR142—exhibited high antioxidant capacity (**Figure 1F**, **Supplementary Table S5**). However, the samples with moderate and low antioxidant capacities lacked clear differentiation. Notably, features such as total antioxidants, FRAP, DPPH, ABTS, alpha-carotene, and beta-carotene displayed relatively high loading scores, contributing significantly to the separation of the samples distinguishing high, medium and low antioxidant activity (**Figure 1G**).

### 3.2 Genetic analysis of lipidome preferentially increased in pigmented sprouts

**Figure 2:**
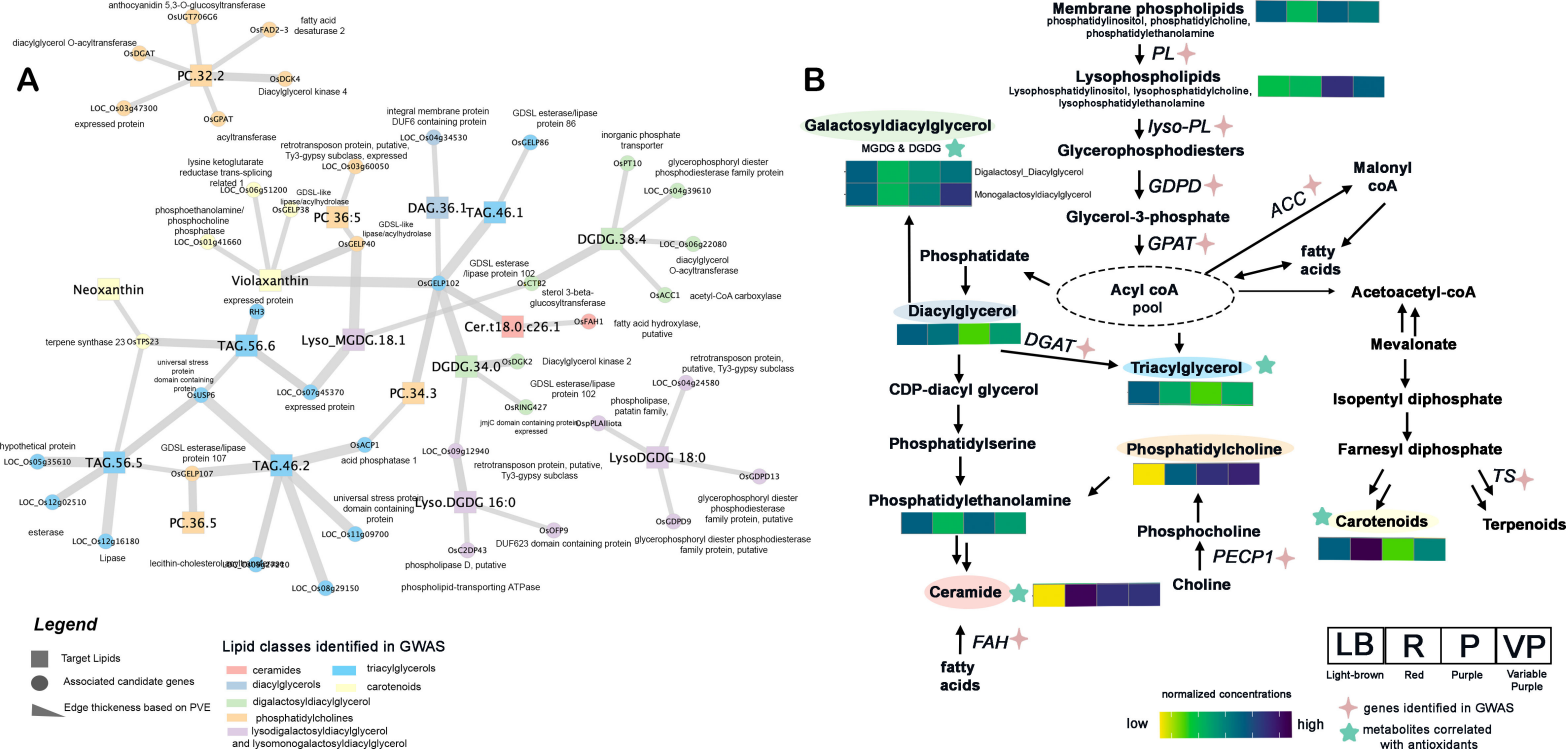
Genetic analysis of lipids in pigmented rice sprouts **(A)** Summary of lipid-candidate genes association with edge connection thickness based on phenotypic variance explained (PVE). **(B)** Proposed summary pathway of lipid metabolism in germinated rice highlighting the lipid-related genes identified in the genome-wide association study (GWAS). PL, phospholipase; GDPD, glycerophosphoryl diester phosphodiesterase; GPAT, glycerol-3-phosphate acyltransferase; ACC, acetyl-CoA carboxylase; DGAT, diacylglycerol acyltransferase; PECP1, phosphoethanolamine/phosphocholine phosphatase; TS, Terpene Synthase; FAH, fatty acid hydroxylase.

Single-locus GWAS revealed 186 SNPs mapped to 174 candidate genes from different chromosomes associated with 20 lipid compounds of PRS based on Bonferroni correction threshold (*P* < 1.00x10^-7^) (**Figure 2**; **Supplementary Figures S4**, **S5**). The gene-level and targeted association analyses narrowed down the list to 72 candidate genes for different lipid compounds (*q-value < 0.01*) (**Table 1**). The combination of single-locus GWAS and targeted association analyses identified 11 candidate genes associated with lipase-related annotations, including six genes encoding GDSL-type esterase/lipase (GELP) proteins (**Supplementary Tables S6**, **S7**).

**Table 1:** SNPs and candidate genes significantly associated with lipids from the single-locus GWAS.

Trait	Chr	Pos	Ref	Alt	p-value	Dist	Candidate genes	Names	Description
Cer.t18.0,c26:1	12	S12_26844342	C	G	5.17E-08	66	LOC_Os12g43363	OsFAH1	fatty acid hydroxylase
DAG.36.1	4	S04_20890828	A	G	6.11E-08	7	LOC_Os04g34530	-	Integral membrane protein DUF6
DGDG 38:4	4	S04_23541915	A	G	4.32E-08	62	LOC_Os04g39610	–	glycerophosphoryl diester phosphodiesterase family protein
DGDG 38:4	5	S05_13080687	G	C	1.28E-08	1	LOC_Os05g22940	OsACC1	acetyl-CoA carboxylase
DGDG 38:4	6	S06_12693175	G	C	8.96E-11	1	LOC_Os06g21950	OsPT10	inorganic phosphate transporter
DGDG 38:4	6	S06_12809964	A	G	8.96E-11	114	LOC_Os06g22080	-	diacylglycerol *O*-acyltransferase
DGDG.34.0	3	S03_12947128	A	T	2.03E-08	1	LOC_Os03g22540	OsRING427	jmjC domain containing protein
DGDG.34.0	9	S09_7463657	G	T	6.33E-08	1	LOC_Os09g12940	-	retrotransposon protein, putative, Ty3-gypsy subclass
Lyso.DGDG16:0	3	S03_12509762	C	T	5.78E-08	1	LOC_Os03g21870	OsOFP9	DUF623 domain containing protein
Lyso.DGDG16:0	5	S05_4389143	G	T	9.73E-08	-128	LOC_Os05g07880	OsC2DP43	phospholipase D, putative
Lyso.DGDG16:0	9	S09_7463657	G	T	4.23E-08	1	LOC_Os09g12940	-	retrotransposon protein, putative, Ty3-gypsy subclass
LysoDGDG 18:0	2	S02_22678979	T	C	8.72E-08	1	LOC_Os02g37590	OsGDPD9	glycerophosphoryl diester phosphodiesterase family protein
LysoDGDG 18:0	4	S04_14121141	A	G	1.58E-09	1	LOC_Os04g24580	-	retrotransposon protein, putative, Ty3-gypsy subclass
LysoDGDG 18:0	9	S09_10079072	C	T	6.72E-08	332	LOC_Os09g17000	OsGDPD13	glycerophosphoryl diester phosphodiesterase family protein
LysoDGDG 18:0	11	S11_23688192	A	G	1.01E-09	168	LOC_Os11g40009	OspPLAIIiota	phospholipase, patatin family
LysoMGDG 8:1	4	S04_1985625	C	G	4.91E-09	1	LOC_Os04g04254	OsCTB2	sterol 3-beta-glucosyltransferase
Neoxanthin	4	S04_16384877	C	T	1.79E-11	1	LOC_Os04g27720	OsTPS23	terpene synthase, putative
PC 32:2	2	S02_29522488	C	T	1.01E-07	70	LOC_Os02g48350	OsDGAT	diacylglycerol *O*-acyltransferase
PC 32:2	3	S03_26757626	T	G	1.76E-12	1	LOC_Os03g47300	-	expressed protein
PC 32:2	5	S05_26226095	T	A	1.95E-08	1	LOC_Os05g45180	OsUGT706G6	anthocyanidin 5,3-*O*-glucosyltransferase
PC 32:2	10	S10_23138938	G	C	4.32E-08	-98	LOC_Os10g42720	OsGPAT	acyltransferase, putative
PC 34:3	1	S01_29972950	T	C	1.13E-09	53	LOC_Os01g52230	OsACP1	phosphoethanolamine/phosphocholine phosphatase
PC 36:5	2	S02_24375062	G	A	1.22E-10	165	LOC_Os02g40440	OsGELP40	GDSL-like lipase/acylhydrolase
PC 36:5	3	S03_34154464	A	C	9.00E-08	1	LOC_Os03g60050	-	retrotransposon protein, putative, Ty3-gypsy subclass
TAG.46.1	6	S06_21353038	A	T	8.09E-08	98	LOC_Os06g36520	OsGELP86	GDSL-like lipase/acylhydrolase, putative
TAG.46.2	8	S08_17922200	T	C	6.65E-08	-71	LOC_Os08g29150	-	phospholipid-transporting ATPase
TAG.46.2	11	S11_5002002	C	G	4.88E-08	176	LOC_Os11g09700	-	anthocyanidin 5,3-O-glucosyltransferase
TAG.46.2	1	S01_36491343	T	A	8.09E-08	1	LOC_Os01g63010	OsUSP6	universal stress protein domain containing protein
TAG.56.5	12	S12_9062378	C	T	1.52E-08	187	LOC_Os12g16180	-	Lipase
TAG.56.5	5	S05_21152663	T	A	5.52E-08	1	LOC_Os05g35610	-	hypothetical protein
TAG.56.5	1	S01_36379718	G	C	1.00E-08	1	LOC_Os01g63010	OsUSP6	universal stress protein domain containing protein
TAG.56.5	2	S12_862041	T	C	1.52E-08	3	LOC_Os12g02510	-	esterase
TAG.56.6	1	S01_36491343	T	A	6.33E-08	1	LOC_Os01g63010	OsUSP6	universal stress protein domain containing protein
TAG.56.6	1	S01_17768946	G	C	2.68E-08	1	LOC_Os01g32380	RH3	expressed protein
TAG.56.6	7	S07_27063835	G	C	6.33E-08	1	LOC_Os07g45370	-	expressed protein
Violaxanthin	1	S01_23488801	C	T	8.12E-08	96	LOC_Os01g41660	-	phosphoethanolamine/phosphocholine phosphatase
Violaxanthin	2	S02_23663747	A	G	8.99E-09	1	LOC_Os02g39170	OsGELP38	GDSL-like lipase/acylhydrolase
Violaxanthin	6	S06_30977509	T	C	1.00E-07	1	LOC_Os06g51200	-	lysine ketoglutarate reductase trans-splicing related 1

#### 3.2.1 Phospholipids and glycerolipids

Seven candidate genes were significantly associated with phospholipids PC 32:2, PC34:3, and 36:5 (**Figure 2A**). The LD block where the peak SNP was located included several candidate genes linked with different phospholipids such as LOC_Os10g42720 (98 kb downstream, *OsGPAT* - acyltransferase, putative), LOC_Os01g52230 (53 kb upstream, *OsACP1* - phosphoethanolamine/phosphocholine phosphatase, putative), and LOC_Os02g40440 (165 kb upstream, *OsGELP40* - GDSL-like lipase/acylhydrolase, putative). In addition, secondary metabolism genes like LOC_Os05g45180 (*OsUGT706G6*, annotated as anthocyanidin 5,3-*O*-glucosyltransferase) showed significant association with PC 32:2 and confirmed by gene-level analysis (*P* < 0.001). Interestingly, Knetminer analysis suggested that UDP glucuronosyltransferases (*UGT*) gene likely to be involved in the lipid glycosylation process in rice (**Supplementary Figure S6**).

Interestingly, TAG 46:2 was linked with the flavonoid-related gene LOC_Os11g09700 (anthocyanidin 5,3-*O*-glucosyltransferase) based on GWAS and gene-level analysis. Other TAGs, such as TAG 46:2, 56:5, and 56:6, were associated with LOC_Os01g63010 (*OsUSP6*, annotated as universal stress protein domain-containing protein), which showed the highest PVE (78.40%) affecting TAG accumulation (**Figure 2A**). Other lipid-related candidate genes associated with TAGs include LOC_Os06g36520 (*OsGELP86*, GDSL-like lipase/acylhydrolase, PVE = 12.98%), LOC_Os08g29150 (phospholipid-transporting ATPase, PVE = 46.7%), LOC_Os12g16180 (lipase, PVE = 46.72%), and LOC_Os12g02510 (esterase, PVE = 16.52%).

#### 3.2.2 Galactolipids

For DGDG 38:4, four candidate genes involved in lipid synthesis were identified from GWAS, namely, LOC_Os04g39610 (glycerophosphoryl diester phosphodiesterase family protein), LOC_Os05g22940 (*OsACC1*), LOC_Os06g21950 (*OsPT10*), and LOC_Os06g22080 (diacylglycerol *O*-acyltransferase) (**Figure 2A**). Among the candidate genes in Chromosome 6, LOC_Os06g22080 (*OsDGAT2*), approximately 114 kb upstream of the top SNPs, is the top candidate gene predicted for DGDG 38:4 and is known to encode for diacylglycerol *O*-acyltransferase. Concurrently, the gene-level analysis verified the *OsDGAT2* with a significant effect association on DGDG 38:4 (p = 8.96x10^-11^). Another gene involved in the upstream processes of the DGDG metabolism that showed significant association is LOC_Os05g22940 (*OsACC1*), annotated as acetyl-CoA carboxylase. In addition, GWAS revealed two candidate genes for DGDG 34:0, namely LOC_Os03g22540 (*OsRING427*) and LOC_Os09g12940 (retrotransposon). Samples possessing the “G” allele for S09_7463657 of LOC_Os09g12940 also showed significantly higher levels of DGDG 34:0 compared to other lines with the alternative allele (**Supplementary Figure S5B**). Among the lyso-galactolipids, significant peaks from Chromosomes 4, 5, and 11 were associated with lysoDGDG 16:0, lysoDGDG18:0, and lysoMGDG 18:1. Interestingly, LD-block analysis revealed phospholipase-encoding genes such as LOC_Os11g40009 (*OspPLAIIiota*, 168 kb upstream) and LOC_Os05g07880 (*OsC2DP43*, 128 kb downstream) were associated with LysoDGDG 18:0 and Lyso.DGDG 16:0, respectively (**Supplementary Figure S7**). In addition, two candidate genes, OsGDPD9 and OsGDPD13, encoding glycerophosphoryl diester phosphodiesterase family proteins were linked with LysoDGDG 18:0. Interestingly, only one candidate gene, LOC_Os04g04254 (*OsCTB2*) which encodes a sterol 3-beta-glucosyltransferase was associated with LysoMGDG 18:1 (β = 2.67) at *P* < 0.001.

#### 3.2.3 Carotenes and sphingolipids

Among the carotenes, neoxanthin, and violaxanthin showed significant associations with candidate genes from Chromosomes 1, 2, 4, and 6 based on GWAS. LOC_Os04g27720 (*OsTPS23*), which encodes a putative terpene synthase, was found to be linked with neoxanthin. The significant SNP (S04_16384877) located upstream of *OsTPS23* showed higher neoxanthin levels for allele “T” over allele “C” (**Figure 2A**, **Supplementary Figure S5B**). On the other hand, violaxanthin was linked with three candidate genes: LOC_Os01g41660, LOC_Os02g39170 (*OsGELP38*), LOC_Os01g41660 (phosphoethanolamine/phosphocholine phosphatase), and LOC_Os06g51200 (lysine ketoglutarate reductase trans-splicing related 1) (**Figure 2A**). Among these, the *OsGELP38* gene, annotated as GDSL-like lipase/acylhydrolase, has a direct role in lipid catabolism. Interestingly, significant allelic variation is found between allele “C” and “T” in S06_30977509 of the LOC_Os06g51200 gene (*P* < 0.001) (**Supplementary Figure S5B**). For sphingolipids, Cer t18:0-c26:1 was predicted to be linked with LOC_Os12g43363 (*OsFAH1*), which is 66 kb upstream of the significant SNPs included in one LD block from Chromosome 12 (p = 5.17x10^-08^).

### 3.3 Genes regulate distinct lipids in PRS linked with antioxidant and anti-cancer properties

Out of 73 unique candidate genes identified from single-locus GWAS and literature mining, the gene-targeted association revealed a total of 11 candidate genes from Chromosomes 1, 2, 4, 7, 8, 9, 10, and 12 possessing significantly associated SNPs to different lipids of PRS (**Table 1**, **Figure 2B**). The candidate genes include: LOC_Os02g40440 (*OsGELP40*), LOC_Os10g05088 (*OsGELP102*), LOC_Os10g30290 (*OsGELP107*), LOC_Os01g52230 (*OsACP1*), LOC_Os04g04254 (*OsCTB2*), LOC_Os04g27720 (*OsTPS23*), LOC_Os07g23410 (*OsFAD2-3*), LOC_Os07g45370 (expressed protein), LOC_Os08g08110 (*OsDGK2*), LOC_Os12g38780 (*OsDGK4*) and LOC_Os09g27210. Two candidate genes of GELP lipase gene family members from Chromosome 10 were associated with different lipids and carotenoids. LOC_Os10g05088 (*OsGELP102*), which possessed multiple intronic and exonic SNPs (synonymous) was significantly associated with DAG 36:1, DGDG 34:0, Cert(18:0/c26:1), and violaxanthin. The LOC_Os10g30290 (*OsGELP107*) gene which possessed different UTR3 and exonic significant SNPs were linked with TAG 46:2, PC 36:5, and TAG 56:5. LOC_Os01g52230 (*OsACP1*) possessed two significant SNPs with non-synonymous mutation (S01_30025718 and S01_30025731) was associated with TAG 46:2. LOC_Os02g40440 (*OsGELP40*), also possessed intronic and exonic SNPs significantly linked with PC 36:5, Lyso_MGDG.18.1, and violaxanthin.

**Figure 3:**
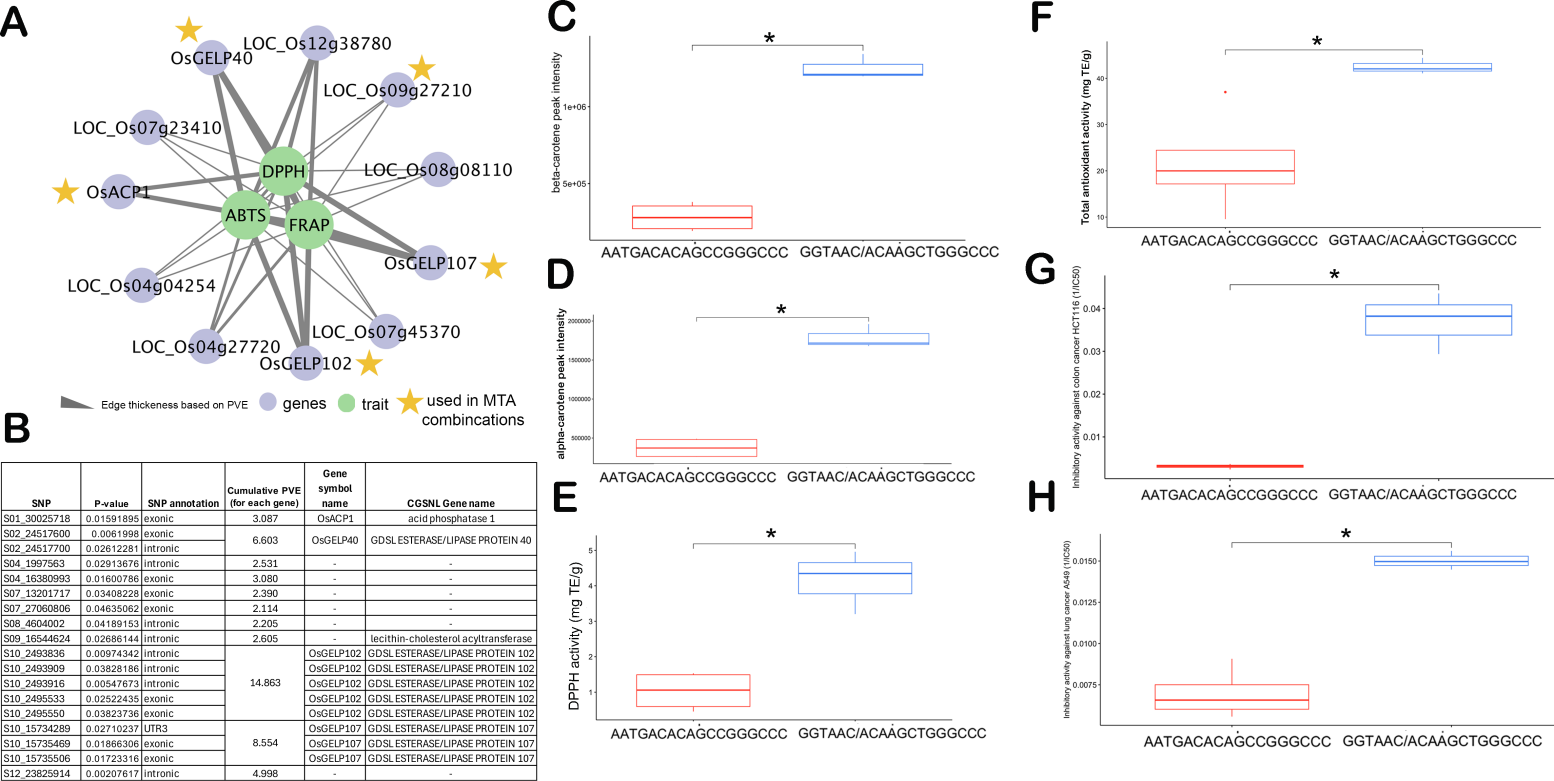
Marker-trait association (MTA) combinations from 18 genes showing contrasting lines (inferior haplotype - AATGACACAGCCGGGCCC vs. superior haplotype - GGTAAC/ACAAGCTGGGCCC) linked with antioxidant and anti-cancer properties. **(A)** network relating the antioxidants with the genes from targeted association. **(B)** Annotation of the top single nucleotide polymorphisms (SNPs) associated with enhanced lipids and antioxidants. Comparison of superior and inferior MTA combinations in terms of: **(C)** beta-carotene, **(D)** alpha-carotene, **(E)** DPPH activity (in mg trolox equivalents/g extract), **(F)** Total antioxidants comprising combinations of DPPH, FRAP, and ABTS (in mg trolox equivalents/g extract), and **(G)** Inhibitory activity against HCT116 colon cancer cell (reported as 1/IC50), **(H)** Inhibitory activity against A549 lung cancer cell (reported as 1/IC50). In the boxplot, the solid middle line depicts the median, while the lower and upper whiskers signify the 25th and 75th percentiles, respectively. The asterisk (*) means significant at p ≤ 0.05.

The high PVE contributing SNPs from top five genes [(LOC_Os02g40440, *OsGELP40*), (LOC_Os10g05088, *OsGELP102*), (LOC_Os10g30290, *OsGELP107*), (LOC_Os01g52230, OsACP1), and (LOC_Os09g27210 - lecithin-cholesterol acyltransferase] identified superior MTA combinations GGTAAC/ACAAGCTGGGCCC exhibiting higher antioxidant activity measured across three independent antioxidant methods, namely 2,2-diphenyl-1-picrylhydrazyl (DPPH), 2,2’-azino-bis(3-ethylbenzothiazoline-6-sulfonic acid (ABTS), and ferric ion reducing antioxidant power (FRAP) (**Figure 3**, **Supplementary Figures S9**, **S10**). Among the antioxidant traits, FRAP demonstrated the highest heritability, ranging from 0.80 to 0.96 (**Supplementary Table S8**). The superior MTA combination (GGTAAC/ACAAGCTGGGCCC) exhibited higher levels of alpha-carotene and beta-carotene as well as the highest DPPH and FRAP antioxidant capacities (**Figure 3**, **Supplementary Figures S9**, **S10**). Interestingly, this finding is consistent with the observation that DPPH is positively correlated with alpha-carotene and beta-carotene levels in the diverse PRS samples (**Figure 1**). To further verify the potential anti-cancer properties, the samples of superior and inferior MTA combinations were taken and tested for their inhibitory activity against colon (HCT116) and lung (A549) cancer cell lines. Interestingly, the superior MTA combinations (GGTAAC/ACAAGCTGGGCCC) possess effective inhibitory activity against HCT116 and A549 with average 1/IC50 of 0.03 and 0.02 (mL/µg) compared to the inferior MTA combination (AATGACACAGCCGGGCCC), respectively (**Figures 3G, H**; **Supplementary Table S9**). It can be surmised that the increase in the carotenoids and unsaturated lipids translate to the antioxidant and anticancer properties in the lipophilic extracts of germinated sprouts.

### 3.4 Selection signals related to OsGELP40, OsGELP102, and OsGELP107 genes

**Figure 4:**
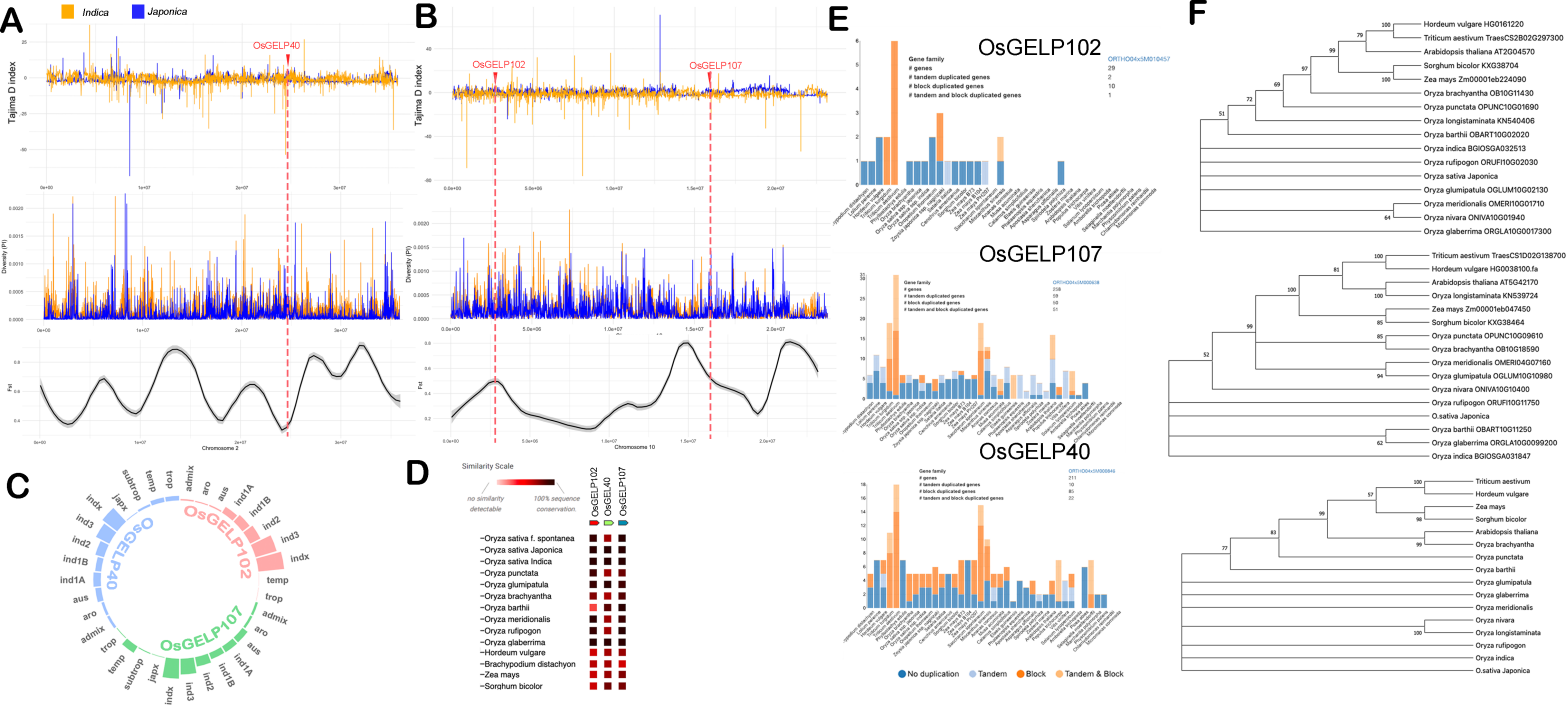
Patterns of selection for the *OsGELP40*, *OsGELP102*, and *OsGELP107* genes measured by Tajima’s D index, nucleotide diversity, and Fixation index (F_ST_). **(A)** *OsGELP40* located on chromosome 2, **(B)** *OsGELP102* and OsGELP107 located on chromosome 10. **(C)** Haplotype distributions linked with *OsGELP40, OsGELP102*, and *OsGELP107* genes using the 3000 rice genome database from the International Rice Research Institute. **(D)** Co-occurrence of *OsGELP40, OsGELP102*, and *OsGELP107* genes in Oryza species and other cereals, **(E)** Gene duplication analysis of the *OsGELP40, OsGELP102*, and *OsGELP107* genes, **(F)** Phylogenetic trees of *OsGELP40, OsGELP102*, and *OsGELP107* genes. GELP - GDSL esterase/lipase.

Genetic analysis provided evidence that the GELP gene family significantly influences lipid composition and increased carotenoid changes in pigmented rice sprouts. To delve deeper, *OsGELP40* (LOC_Os02g40440), *OsGELP102* (LOC_Os10g05088), and *OsGELP107* (LOC_Os10g30290) lipase genes were scrutinized to determine if they experienced selection pressures during pigmented rice domestication. Selection scans on three lipase genes located on chromosomes 2 and 10 were conducted through analysis of Tajima’s D index (**Figure 4A**) and F_ST_ values (**Figure 4B**) using pigmented rice *japonica* and *indica* subspecies. OsGELP40 exhibited positive Tajima’s D indices for both *indica* (0.79) and *japonica* (2.26), coupled with a relatively low F_ST_ value of 0.07, indicative of balancing selection that contributes to maintaining genetic diversity within both populations. Conversely for *OsGELP102* gene, *Japonica* displayed a positive Tajima’s D (3.14), while *Indica* exhibited a negative Tajima’s D (-2.48), suggesting potential demographic events or selection processes leading to distinct patterns of genetic diversity. Additionally, a F_ST_ value of 0.397 indicated substantial genetic differentiation between *Japonica* and *Indica*. Similar patterns were observed in *OsGELP107* concerning Tajima’s D and F_ST_. The expression of *OsGELP40*, *OsGELP102* and *OsGELP107* were found in germinating sprouts (**Supplementary Figure S12**).

The 3000 rice genome database was queried for superior alleles associated with these genes. Superior alleles linked to *OsGELP40* exhibited distribution across rice subspecies, with *Indica* having the highest percentage. Conversely, *OsGELP102*-related superior alleles were exclusively found in *Indica*, not in *Japonica* (**Figure 4C**). Similar observations were made for *OsGELP107* haplotypes, with a small percentage present in temperate Japonica. **Figure 4D** revealed conserved gene sequences of these GELP genes in *O.sativa, O.glumipatula*, and *O.glabberima*. Gene duplications were analyzed across species revealing non-duplicated and block duplications for OsGELP40 in both *Indica* and *Japonica*. Interestingly, different patterns for *OsGELP102* gene were observed, where in tandem and block duplications were found in *Indica* and no duplications noted in *Japonica* (**Figure 4E**). For OsGELP107 no duplications were observed in either subspecies. In the phylogenetic analysis involving wild rice species, it was observed that there is no distinct divergence between domesticated rice and wild rice species (**Figure 4F**).

## 4 Discussion

### 4.1 Preferential enrichment of carotenoids and lipids in pigmented rice sprouts contribute to antioxidant and anti-cancer property

Although secondary metabolites are predominantly responsible for the coloration of PRS (Tiozon et al., 2021), our study showed that significantly higher proportions of unsaturated DAGs and TAGs were present in PRS compared to non-pigmented samples. Moreover, substantial accumulations of carotenoids and unsaturated lipids under the classification of TAGs, DGDGs, and PCs were observed in the red and purple variants compared to light-brown rice sprouts, while higher carotenoids were observed in purple rice than in red rice. Notably, the fatty acids present within both DAGs and TAGs have been found to exhibit a heightened antioxidant capacity (Khan et al., 2019). Primarily, unsaturated fatty acids can readily donate electrons to unstable free radicals (Khan et al., 2019). Notably, *GELP* genes such as LOC_Os10g30290 (*OsGELP107*), LOC_Os10g05088 (*OsGELP102*), and LOC_Os01g61200 (*OsGELP26*) have been associated with triglycerides such as TAG 46:1, TAG 46:2, and TAG 56:5 (**Figure 2**; **Supplementary Figure S11**). Interestingly, these segmentally duplicated genes belong to the same phylogenetic subclade (Chepyshko et al., 2012), parallel to the current findings, which identified these genes to be linked with TAGs of similar chemical structures. Furthermore, few of the galactolipids (DGDG 38:4, DGDG 36:4, and MGDG 36:4) have correlation with antioxidants (**Figure 1E**). GWAS revealed associations of DGDG 38:4 with four candidate genes: LOC_Os04g39610, LOC_Os05g22940 (*OsACC1*), LOC_Os06g21950 (*OsPT10*), and LOC_Os06g22080 (*OsDGAT2*) (**Figure 2**). One of the top candidate genes, *OsDGAT2*, confirmed through both GWAS and gene-level analysis, is known to encode for a diacylglycerol *O*-acyltransferase is involved in the key rate-limiting step in the conversion of DAGs into TAGs through the esterification of the third acyl chain of DAG (Bhunia et al., 2021). *OsACC1* gene linked with DGDG 38:4 functioning upstream of DGDG synthesis plays a crucial role in fatty acid synthesis and metabolism (Tiwari et al., 2016; Bhunia et al., 2021).

In general, carotenoids are known for their photoprotective functions by dissipating excess light contributing to scavenge free radicals, and inhibit lipid peroxidation (Rodriguez-Concepcion et al., 2018). Concurrently, Pereira-Caro et al. (2013) revealed higher total carotenoid content was observed in rice samples with black pigmentation compared to red-colored rice. In plants, the relationship between lipids and the carotenoid pathway is complex yet interconnected. Lipids play a crucial role in providing the structural components and energy required for carotenoid biosynthesis, storage, and function. Additionally, lipids can act as carriers and protectors of carotenoids within the plant cells (Barja et al., 2021). Both lipids and carotenoids share common precursor molecules, namely isopentenyl diphosphate (IPP) and dimethylallyl diphosphate (DMAPP), which are derived from the mevalonate (MVA) or the methylerythritol phosphate (MEP) pathways. Furthermore, geranylgeranyl pyrophosphate (GGPP), a precursor for carotenoid biosynthesis, is synthesized from IPP and DMAPP, which are derived from the lipid pathway (Barja et al., 2021). GGPP is a crucial precursor for carotenoids, and its availability is closely linked to lipid metabolism. Compared to other carotenoids like β-carotene and lutein, the levels of neoxanthin in seeds are relatively lower, yet its presence could play a role in enhancing the nutritional value of rice as a dietary source of carotenoids (Saini et al., 2022). GWAS analysis revealed that *OsTPS23* (LOC_Os04g27720), which encodes for terpene synthase is significantly linked with neoxanthin. Protein-protein network analysis shows that terpene synthase gene is interacting with geranylgeranyl diphosphate synthase (GGDPS) (**Supplementary Figure S8**), an enzyme that plays a key role in the biosynthesis of carotenoids in plants. Mechanistically, GGDPS catalyzes the formation of GGPP from farnesyl diphosphate in the plastidic isoprenoid biosynthetic pathway. GGPP is used as a precursor for the synthesis of various carotenoids, which are important for light harvesting and photoprotection in plants (Barja et al., 2021). It can be inferred that PRS potentially possesses higher levels of lipid intermediates, enabling the accumulation of increased carotenoids and lipids.

The lipidome-GWAS identified the importance of *GELP* genes relating to the elevation in alpha and beta carotenoids in the present study. The superior alleles combined from MTAs (GGTAAC/ACAAGCTGGGCCC) of top five genes including three GELP gene family members exhibits the highest levels of alpha- and beta-carotene, which corresponds to its high DPPH and overall antioxidant activity (**Figure 3**). Furthermore, the pigmented donor lines exhibiting the GGTAAC/ACAAGCTGGGCCC haplotype represent significant donor candidates due to their consistently elevated antioxidant activity and anticancer property.

Lipases are attributed to the degradation of lipids resulting in complex fatty acids (Howitt and Pogson, 2006). In particular, TAG lipases play a crucial role in lipid homeostasis by breaking down TAGs into free fatty acids and glycerol. These enzymes are involved in various physiological processes, including energy mobilization, signal transduction, and stress responses (Howitt and Pogson, 2006). In addition, the generation and availability of free fatty acids are likely influential factors in the esterification and accumulation of carotenoids. Mechanistically, during germination, it is possible that carotenoids that are stored in lipid droplets during seed maturation are mobilized by lipases upon seed germination that undergo esterification with fatty acids, initiating the potential mobilization of the triglycerides and carotenoids (Abreu et al., 2020). Likewise, observations in other species have highlighted that owing to the lipophilic nature of carotenoids, they can be stored in lipid droplets. Subsequently, these carotenoids may be cleaved by lipases, facilitating their further mobilization and consequently enhancing detectable carotenoid levels (Abreu et al., 2020; Bu et al., 2022). Furthermore, lipids and phenolic compounds are also known to interact in plants to influence color and bioactivity (Karonen, 2022).

The accumulation of lipids can be associated with lipophilic antioxidant capacities, thus potentially representing a novel dietary source for human health. In fact, **Figure 1** shows that there is an increased antioxidant capacity of lipophilic fractions after germination. Particularly, PRS with purple and variable purple color showed important donor lines with enhanced antioxidant activities of lipophilic fractions after the germination process. Similarly, Mohd Esa et al. (2013) demonstrated that germinated brown rice has higher lipid antioxidant enzyme activities such as superoxide dismutase and glutathione peroxidase compared with brown and milled rice (Mohd Esa et al., 2013). Lipophilic antioxidants, including lipophilic vitamins and unsaturated fatty acids, exhibit high effectiveness in protecting cell membranes from lipid oxidation. These antioxidants play a crucial role in preventing the oxidative damage of cell membranes by scavenging reactive oxygen species and inhibiting lipid peroxidation processes (Lagouri, 2014). Concurrently, alpha- and beta-carotene, as well as triglycerides with higher degrees of unsaturation, such as TAG 56:6 and 56:5 and DGDG 38:4 demonstrated moderate correlations with certain antioxidant tests (**Figure 1**). Mechanistically, carotenoids operate through the presence of a conjugated system, wherein the π-electrons are efficiently delocalized across the entire length of the polyene chain. This delocalization enables carotenoids to exhibit unique antioxidant properties, making them important contributors to rice’s dietary properties (Lagouri, 2014). In fact, clinical studies have demonstrated that carotenoids engage in cross-talk with other lipids in the human body, thereby promoting enhanced immune system functions and lowering the risk of developing degenerative chronic diseases (Rodriguez-Concepcion et al., 2018; Marhuenda-Muñoz et al., 2022). These diseases include but are not limited to age-related macular degeneration, type 2 diabetes, obesity, certain types of cancer (breast, cervical, ovarian, colorectal), and cardiovascular diseases (Rodriguez-Concepcion et al., 2018; Marhuenda-Muñoz et al., 2022). Consistent with this feature, carotenoids are known to regulate the hallmarks of cancers through immune modulation, hormone and growth factor signaling, regulatory mechanisms of cell cycle progression, cell differentiation and apoptosis (Niranjana et al., 2015). The anti-cancer effect of carotenoids may be associated with the generation of reactive oxygen species, triggering cytotoxicity and apoptosis which involves the cleavage of caspases-3 and -9, as well as poly-ADP-ribose polymerase, accompanied by reductions in the levels of Bcl-xL (Sathasivam and Ki, 2018). Furthermore, polyunsaturated fatty acids may work through the antioxidant signaling pathway regulation and inflammatory cycle modulation (Calder, 2020). The enhanced antioxidant properties of the lipophilic fraction enriched with carotenoids in PRS in the diet may be beneficial to potentially promote human health by reducing the risks of inflammation, cardiovascular disorder, and cancer (Lagouri, 2014; Calder, 2020).

### 4.2 Differential selection signals associated with OsGELP102 and OsGELP107 in *Indica* and *Japonica* subspecies, and the potential roles of these genes in antioxidant properties

The phylogenetic tree of the *OsGELP* gene family suggests that, *OsGELP40, OsGELP102*, and *OsGELP107* were recognized to occupy the same clade (Chepyshko et al., 2012). Notably, *OsGELP102* and *OsGELP107* genes, exhibiting distinct Tajima’s D indices and relatively high F_ST_ values, were further identified to be situated on a common subclade (Chepyshko et al., 2012). Besides on their role of lipid mobilization, these GELP genes were also found to be responsive to both biotic and abiotic stress (Yao-Guang et al., 2022). In addition, experimental evidences revealed that these genes may also influence the secondary metabolism pathways, plant development, and morphogenesis (Chepyshko et al., 2012). The accumulation in the lipid metabolites in pigmented rice sprouts that translated to its antioxidant capacity can potentially be attributed to these *GELP* genes. Interestingly, the GELP genes catalyze the esterification of an antioxidant carotenoid namely lutein in wheat (Watkins et al., 2019). These findings underscore the role of these genes in enhancing the antioxidant and anticancer activity of cereals.

From an evolutionary perspective, *GELP*s play an important role in cuticle formation, particularly cutin and suberin, which is crucial for the terrestrial colonization of plants (Shen et al., 2022). Recently, Jing et al. (2023) revealed 30 genes associated with domestication in rice where many of these genes are associated with plant growth and response to stress (Jing et al., 2023). Moreover, these *GELP*s play roles in these functions, prompting an analysis of population genomic parameters such as F_ST_ and Tajima’s D index in pigmented rice samples of *Indica* and *Japonica* which unveils potential differences in their selection process. The negative Tajima’s D index for the *OsGELP102* and *OsGELP107* genes could be indicative of population expansion, positive selection, or purifying selection in *Indica*. Furthermore, the relatively high F_ST_ value suggests strong genetic differentiation between *Japonica* and *Indica* populations, indicating limited gene flow between them linked to these genes. In fact, recent genomic and archaeological discoveries indicate that the initiation of *indica* domestication may have occurred approximately 8,000 years before the present, significantly earlier than the previously speculated timeframe thus suggesting that domestication was likely in progress in northern India before the arrival of *japonica* (Bates et al., 2017; Jing et al., 2023). It is noteworthy to further scrutinize the selection signals associated with the *GELP* genes.

## 5 Conclusion

Rice, as a crucial staple food supplying the primary caloric intake for over half of the global population, offers immense potential for nutritional enhancement through innovative bioprocessing techniques. Germination, in particular, emerges as a promising method to boost the nutrient profile of rice, more so to explore PRS as novel source of future functional foods making it more beneficial for health. Genome-wide association studies (GWAS) have identified GELP genes that play a pivotal role in mobilizing lipids and carotenoids during germination. This mobilization has been linked to enhanced antioxidant properties and significant anti-cancer activities, underscoring the potential health benefits of germinated rice. This finding highlights the need for further investigations to explore the roles of GELP genes in rice domestication and their functional significance in lipid mobilization. Functional validation of these genes could pave the way for developing rice varieties with higher concentrations of bioactive lipids, carotenoids, and other health-promoting compounds. Nevertheless, these insights offer an opportunity to identify genetic markers that could guide the breeding of nutrient-dense, pigmented rice sprouts, which aligns with global efforts to combat malnutrition by introducing rice varieties enriched with essential nutrients and bioactive compounds.

## Data Availability

The original contributions presented in the study are included in the article/**Supplementary Material**, further inquiries can be directed to the corresponding author/s.
