# Supplementary material for: Lipidomics-based association study reveals genomic signatures of anti-cancer qualities of pigmented rice sprouts
Source: Front Plant Sci. 2025 Jan 28;16:1533442. doi: 10.3389/fpls.2025.1533442 (PMC11810972; doi:10.3389/fpls.2025.1533442)
Supplement: Supplementary file 2 [file DataSheet1.pdf]

Supplementary Figures

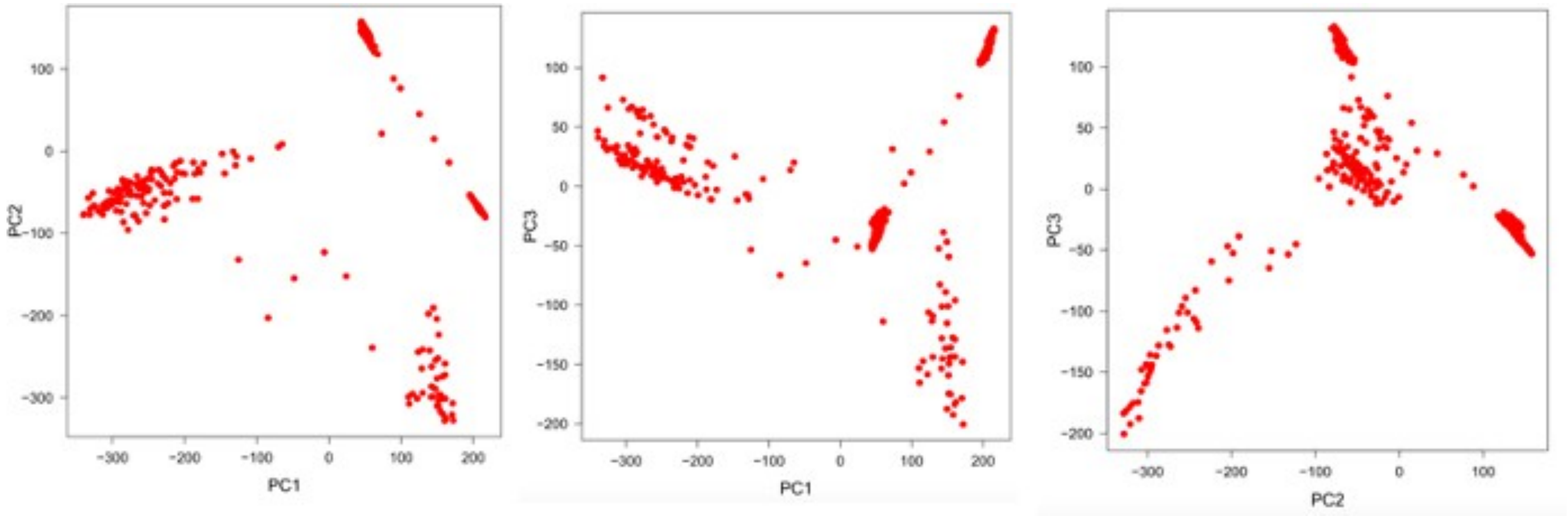

Figure S1. Principal Component Analysis plots used in this study

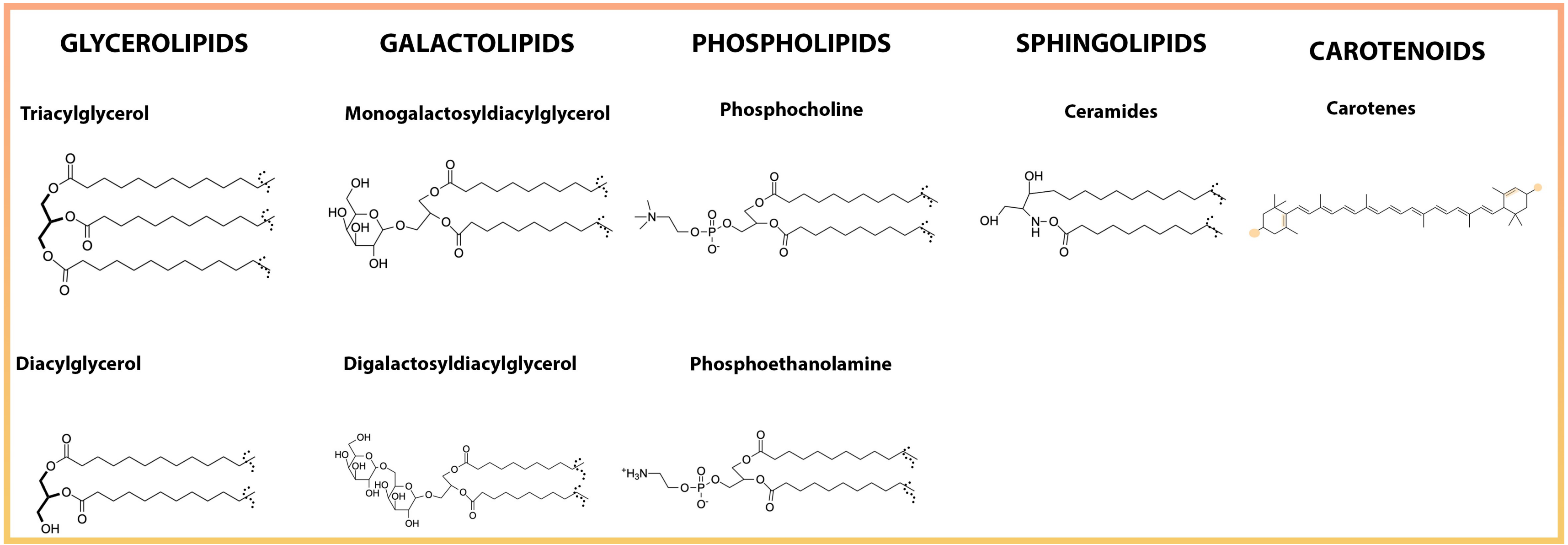

Figure S2. General structures of the lipids

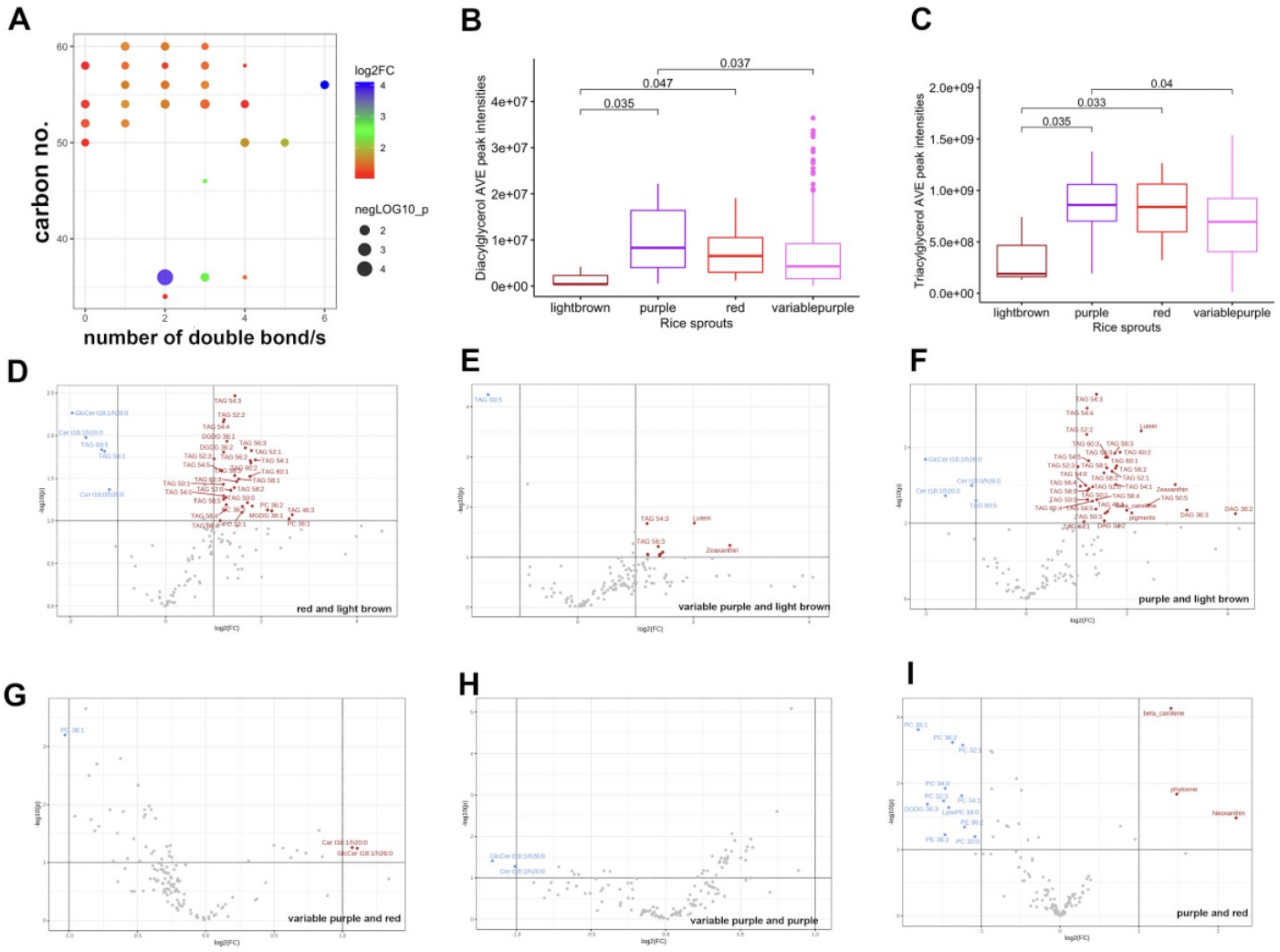

Figure S3. Lipid variation based on rice sprouts' colors (A) fold-change in pigmented and non-pigmented rice based on the carbon length and degree of unsaturation, (B) average peak intensities of diacylglycerols, (C) average peak intensities of triacylglycerols. In

the boxplot, the solid middle line depicts the median, while the lower and upper whiskers signify the 25th and 75th percentiles, respectively. Volcano plot comparing rice sprouts with colors (D) red and light brown, (E) variable purple and light brown, (F) purple and light brown, (G) variable purple and red, (H) variable purple and purple, (I) purple and red

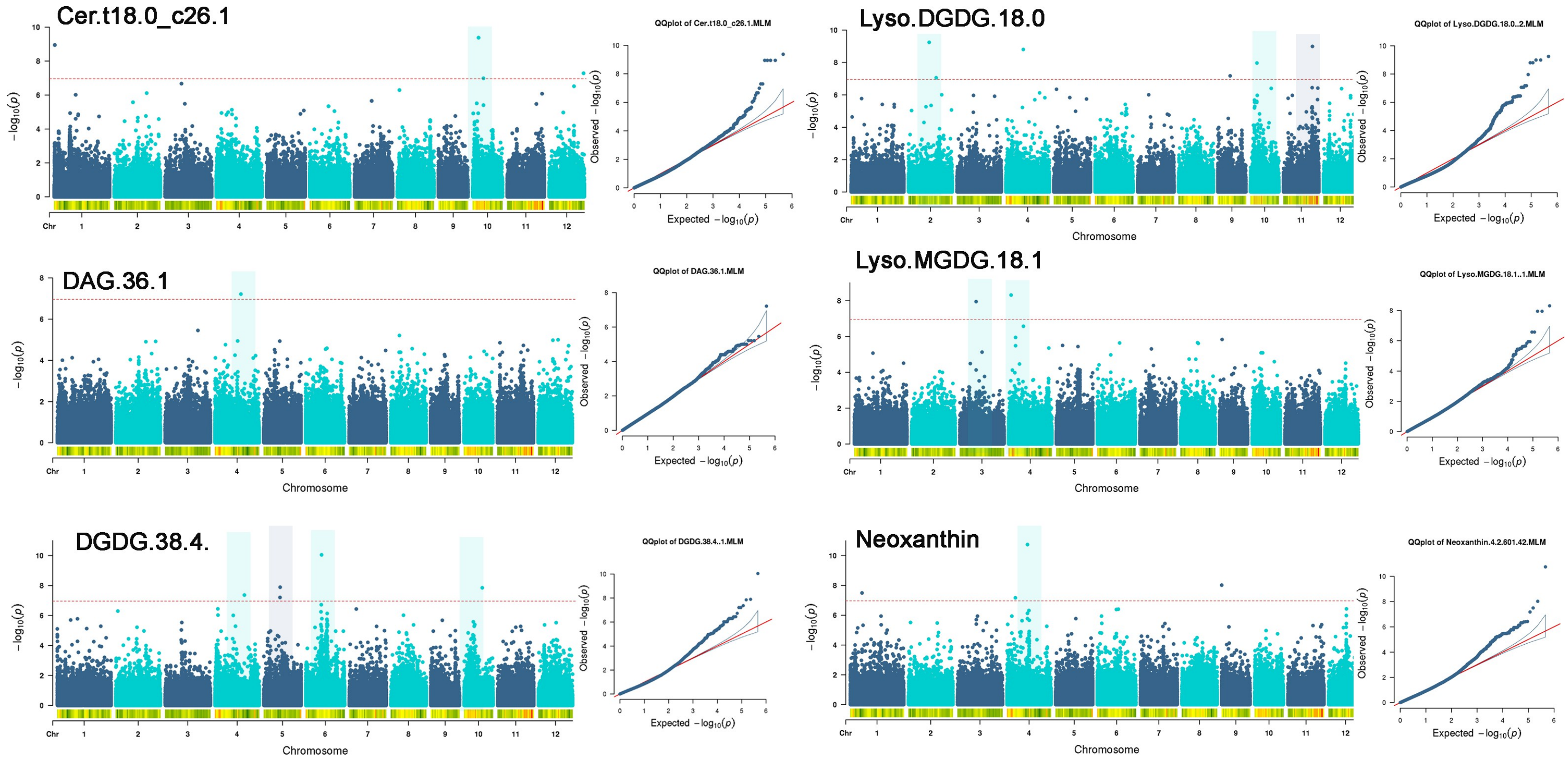

Figure S4. Representative Lipidome-genome wide association study plots from this study

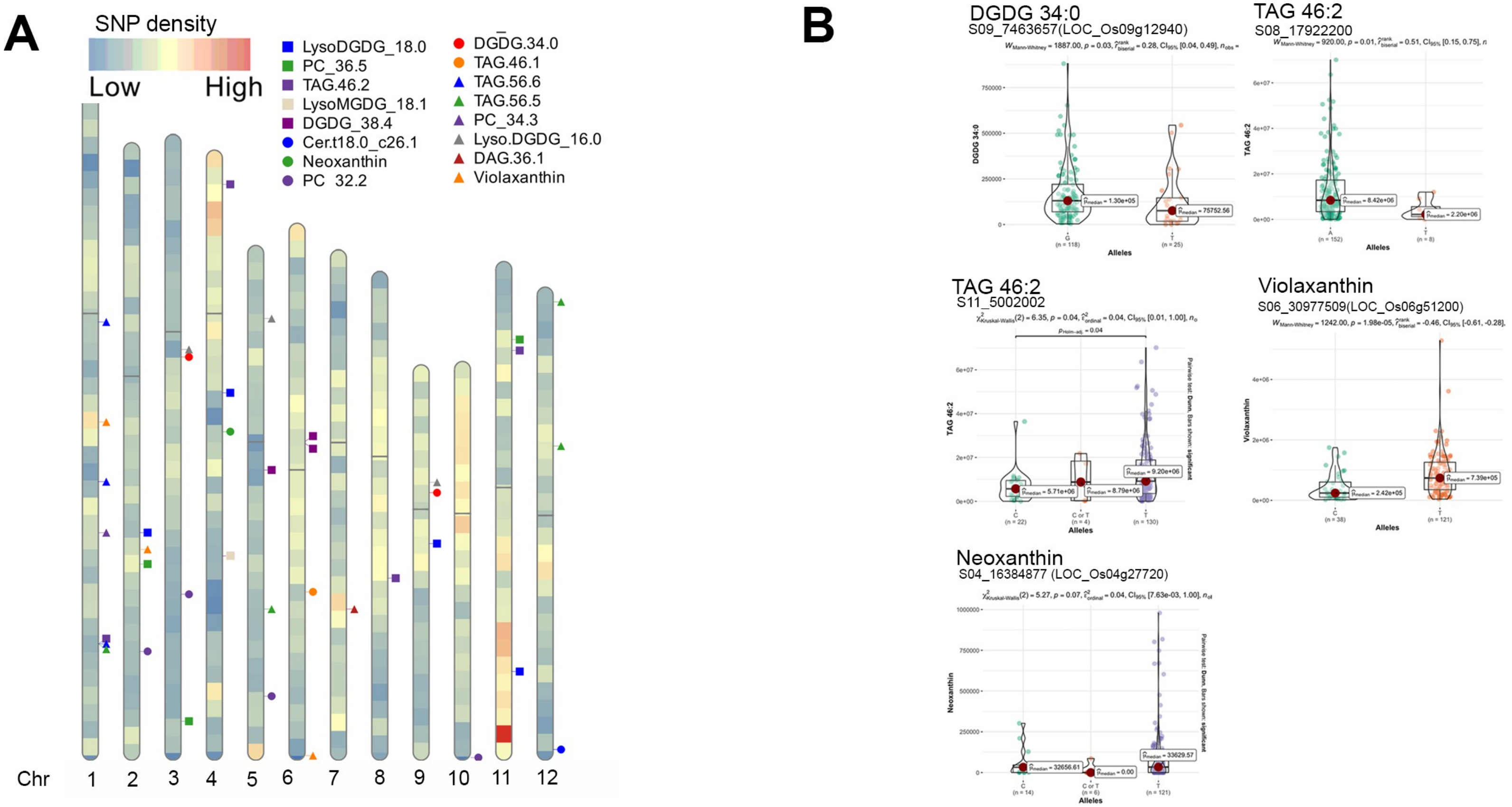

Figure S5. (A) Chromosomal locations of the candidate genes associated with lipids based on single-locus GWAS and linkage analysis. Colored symbols beside each chromosome show the corresponding lipid compounds associated to the identified candidate genes (C) Allelic boxplot of the selected significant SNPs associated with corresponding lipids

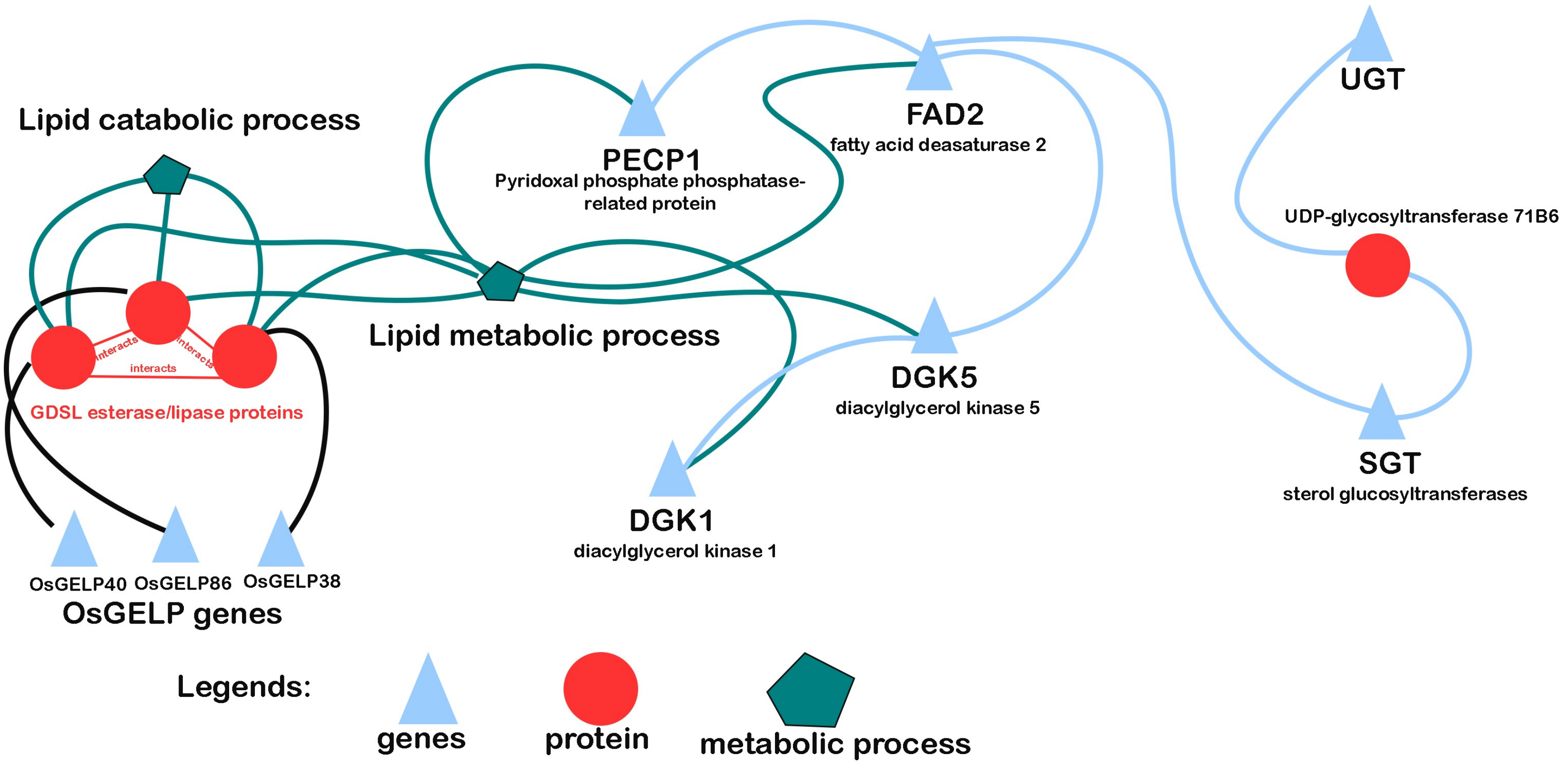

Figure S6. The gene network summary for genes in the targeted association analysis. Abbreviations: OsGELP - *Oryza sativa* Glycosyltransferase-like Enzyme, FAD - Fatty Acid Desaturase, DGK - Diacylglycerol Kinase, PDAT - Phospholipid:Diacylglycerol Acyltransferase, UGT - UDP-Glycosyltransferase

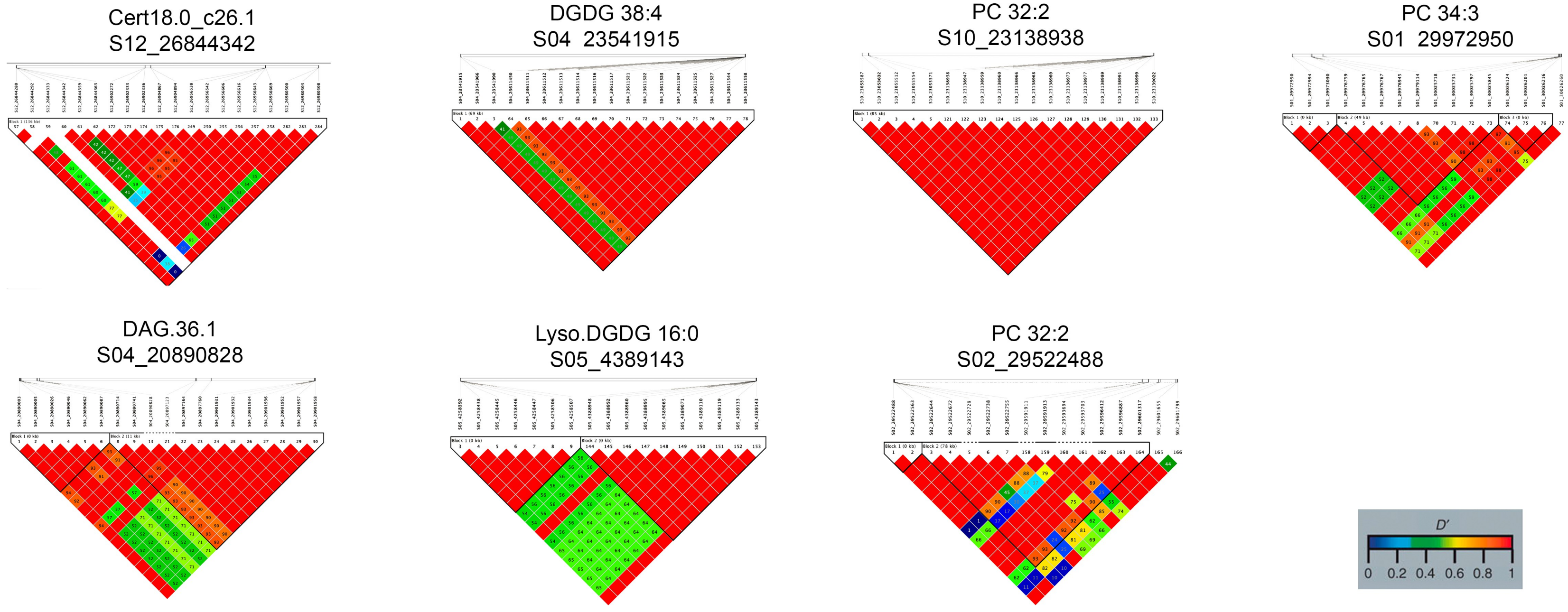

Figure S7. The linkage disequilibrium of the top SNPs from the GWAS results

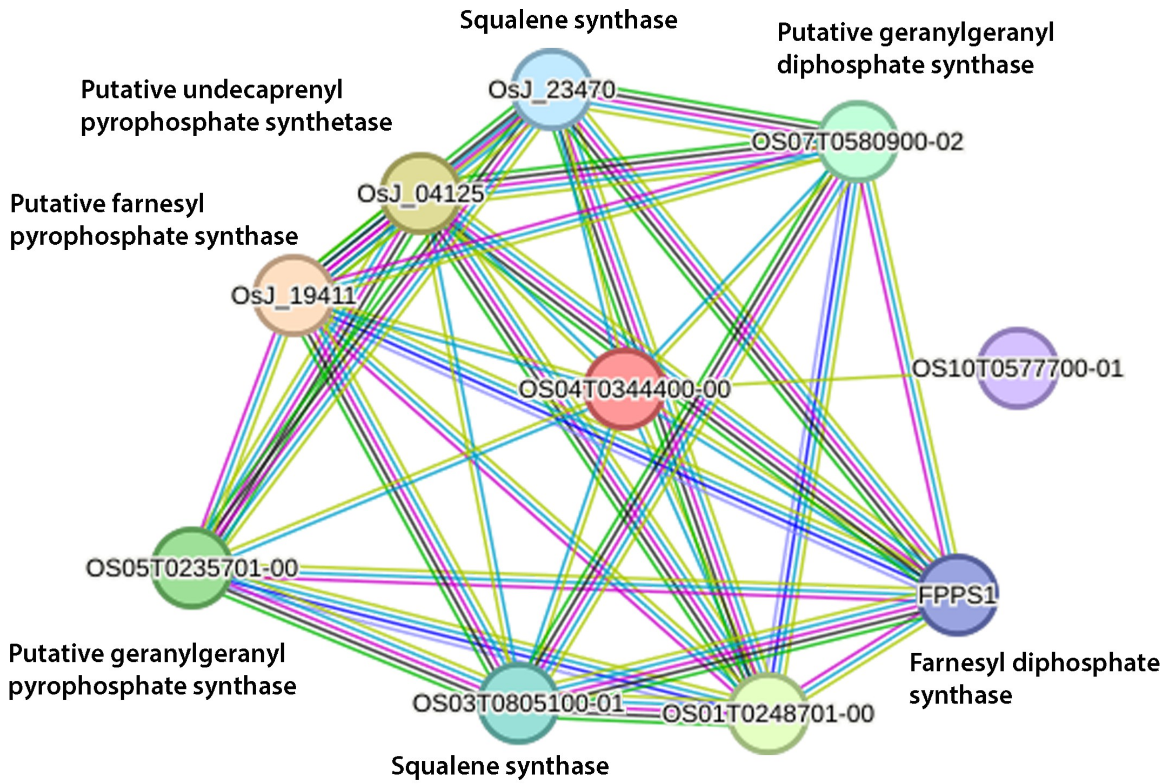

Biological Process (Gene Ontology)

GO-term	description	count in network	strength	false discovery rate
GO:0033386	Geranylgeranyl diphosphate biosynthetic process	3 of 3	3.55	1.31e-07
GO:0033384	Geranyl diphosphate biosynthetic process	5 of 7	3.41	6.00e-13
GO:0045338	Farnesyl diphosphate metabolic process	7 of 15	3.22	8.11e-18
GO:0045337	Farnesyl diphosphate biosynthetic process	5 of 11	3.21	1.91e-12
GO:0006695	Cholesterol biosynthetic process	2 of 5	3.15	0.00026

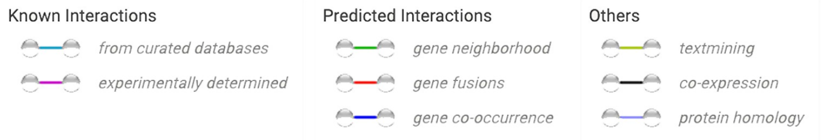

Figure S8. Protein-protein interaction between the terpene synthase gene and other carotenoid-related genes

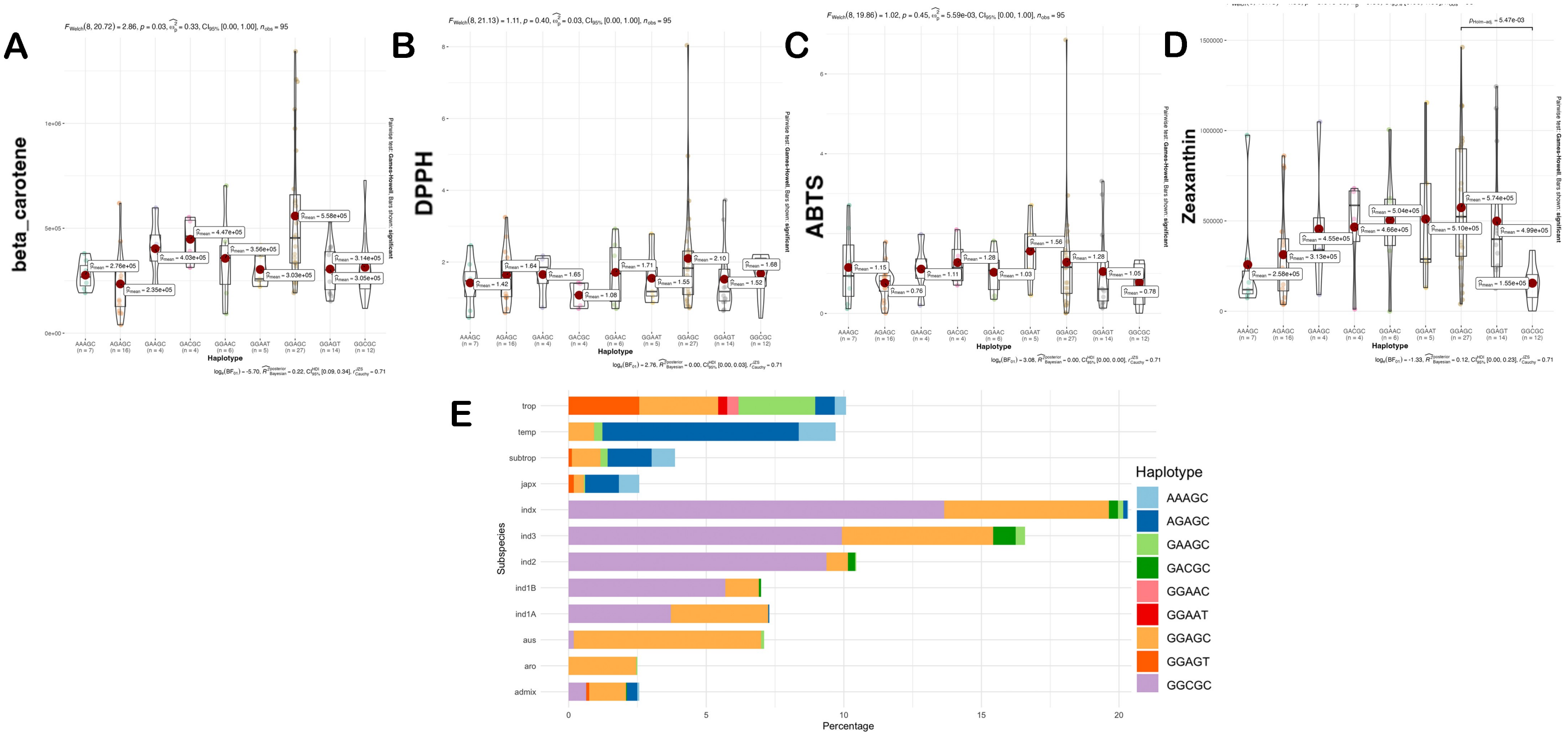

Figure S9. Marker-trait association (MTA) combinations using the top 5 snps from the targeted association and mining the 3000 rice genome database for these haplotypes

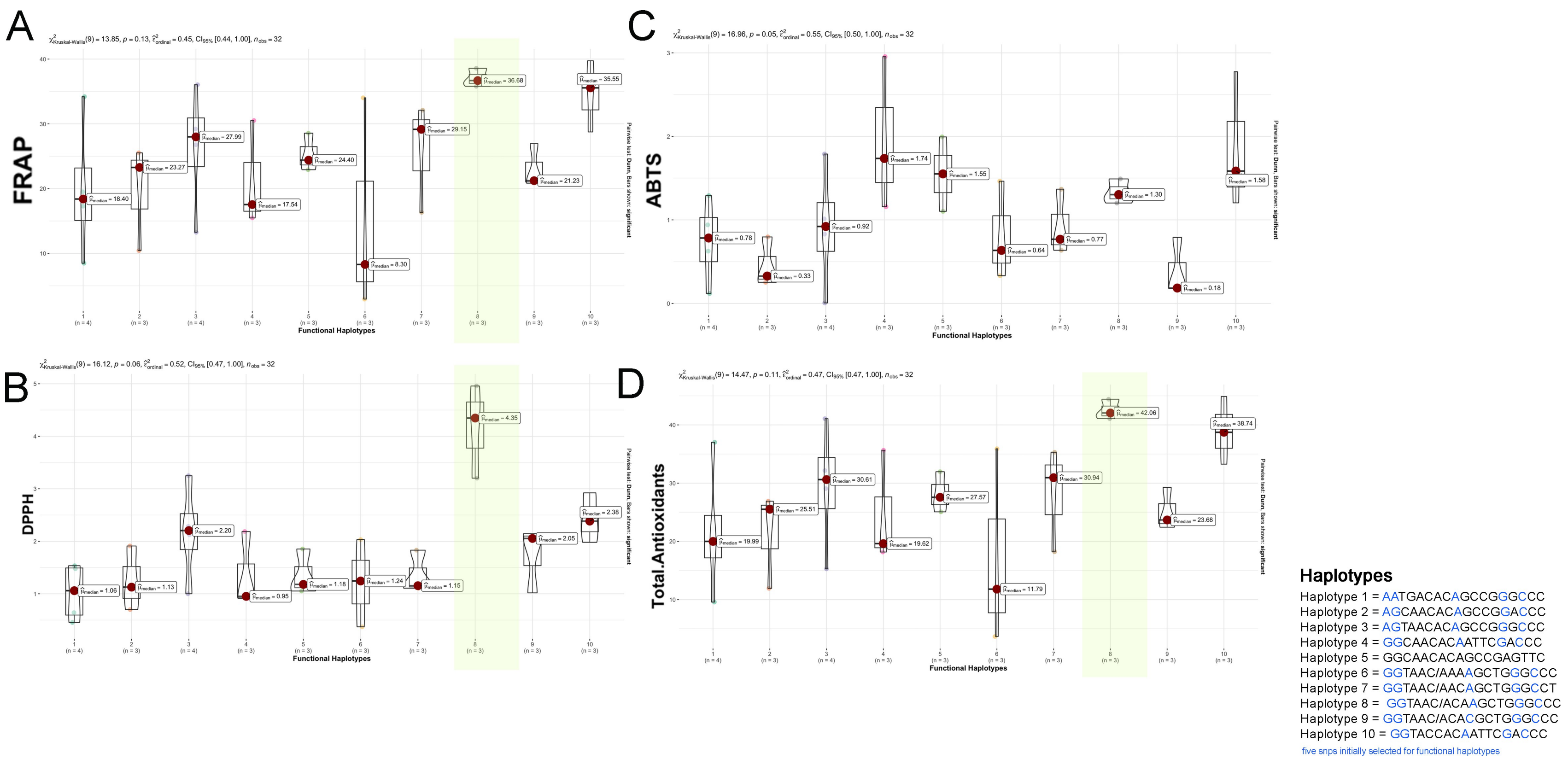

Figure S10. MTA combinations using 18 snps that are linked to the antioxidant capacity. In the boxplot, the solid middle line depicts the median, while the lower and upper whiskers signify the 25th and 75th percentiles, respectively.

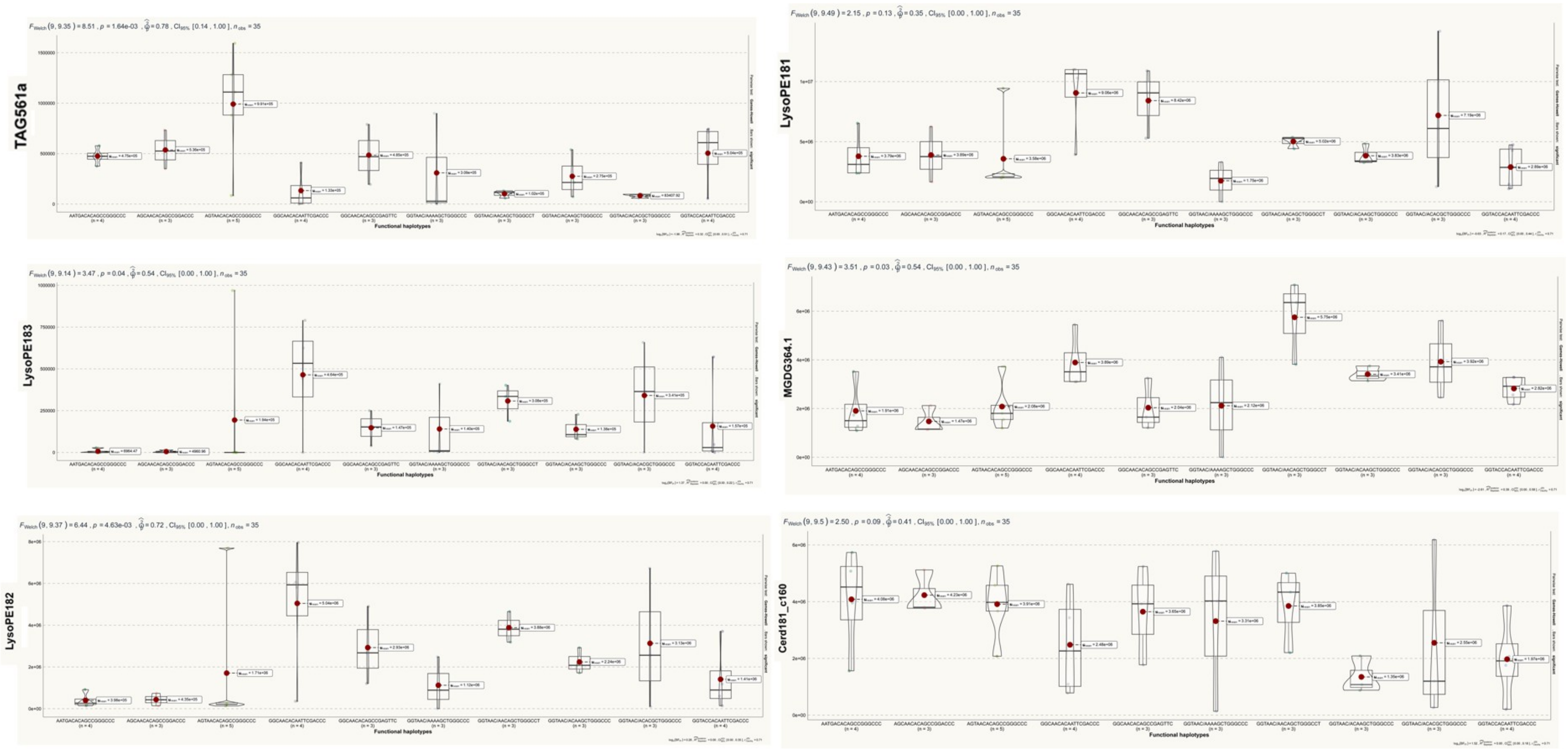

Figure S11. Functional haplotypes using 18 snps that are linked to the lipid-related compounds. In the boxplot, the solid middle line depicts the median, while the lower and upper whiskers signify the 25th and 75th percentiles, respectively.

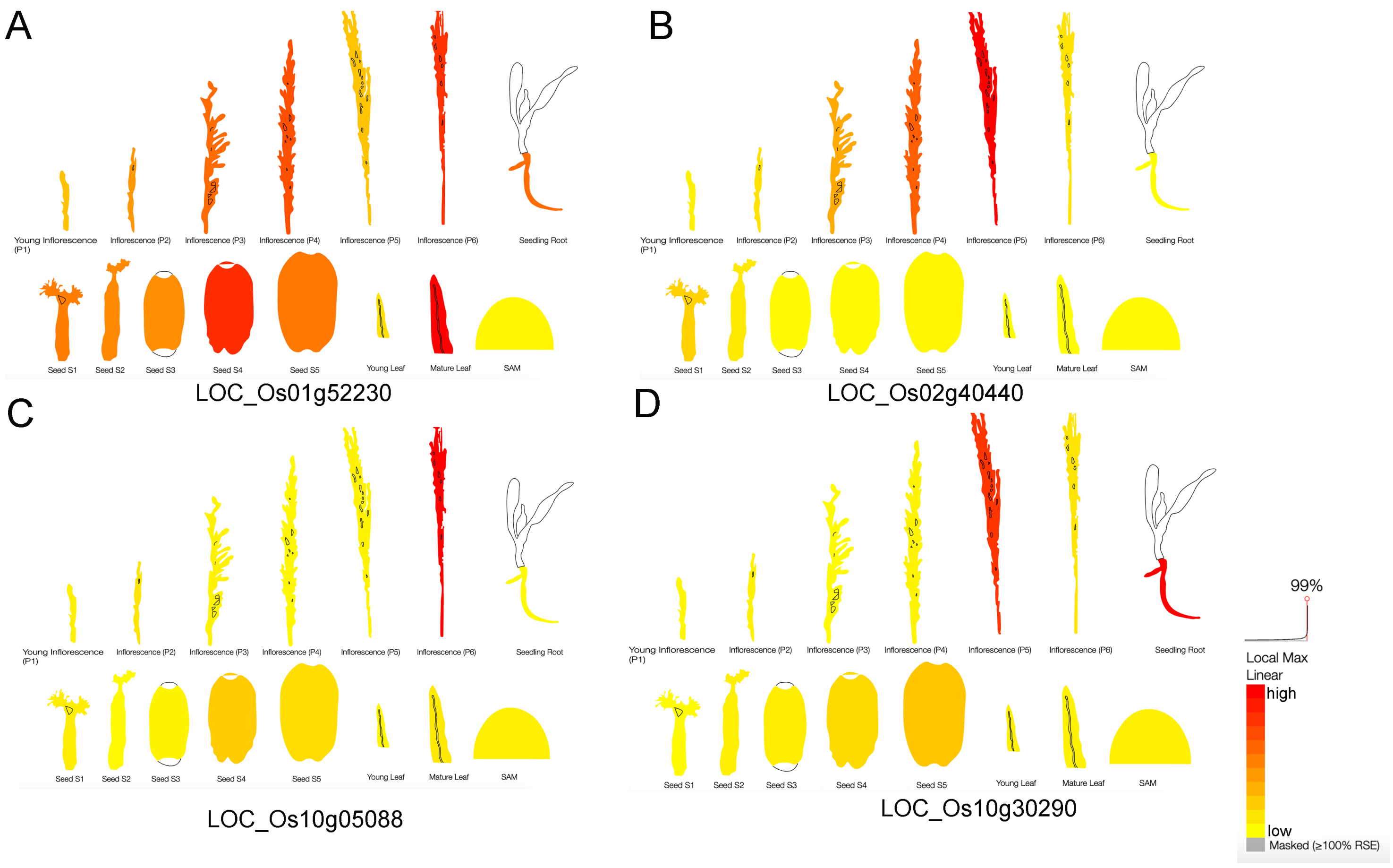

Figure S12. Expression analysis of high PVE conferring genes linked to mobilization of lipids and antioxidant properties.(A) LOC_Os01g52230: OsACP1, acid phosphatase 1; (B) LOC_Os02g40440: OsGELP40, GDSL ESTERASE/LIPASE PROTEIN 40; (C) LOC_Os10g05088: OsGELP102, GDSL ESTERASE/LIPASE PROTEIN 102; (D) LOC_Os10g30290: OsGELP107, GDSL ESTERASE/LIPASE PROTEIN 107. Data were taken from https://bar.utoronto.ca/eplant_rice/
